# New amide alkaloids from *Piper longum* fruits

**DOI:** 10.1007/s13659-013-0073-0

**Published:** 2013-11-27

**Authors:** Jun Yang, Yao Su, Ji-Feng Luo, Wei Gu, Hong-Mei Niu, Yan Li, Yue-Hu Wang, Chun-Lin Long

**Affiliations:** 1 Key Laboratory of Economic Plants and Biotechnology, Kunming Institute of Botany, Chinese Academy of Sciences, Kunming, 650201 China; 2 College of Resources and Environmental Sciences, Hebei Agricultural University, Hebei, 071000 China; 3 State Key Laboratory of Phytochemisty and Plant Resources in West China, Kunming Institute of Botany, Chinese Academy of Sciences, Kunming, 650201 China; 4 College of Life and Environmental Sciences, Minzu University of China, Beijing, 100081 China

**Keywords:** Piperaceae, *Piper longum*, amide alkaloids, piperlongumamides, piperchabamide B, cytotoxicity

## Abstract

Three new amide alkaloids piperlongumamides A-C (**1–3**), together with 12 known ones (**4–15**), were isolated from the fruits of *Piper longum*. The structures of the new isolates were determined using spectroscopic data analyses. Cytotoxic activity of these amides against HL-60 (human leukemia), A-549 (human lung cancer), MCF-7 (human breast cancer), SMMC-7721 (human liver cancer) and SW480 (human rectal cancer) cell lines were evaluated. Piperchabamide B (**11**) exhibited weak inhibitory activity against HL-60 (IC_50_ = 21.32 μM ), A-549 (IC_50_ = 23.82 μM ) and MCF-7 (IC_50_ = 16.58 μM ) cell lines.

**Electronic Supplementary Material:**

Supplementary material is available for this article at 10.1007/s13659-013-0073-0 and is accessible for authorized users.

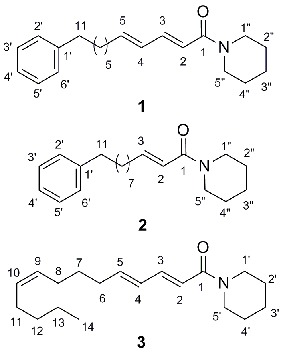

## Introduction

*Piper longum* L. (Piperaceae) is a slender aromatic climber with perennial woody roots which grows primarily in tropical regions. Its fruits and roots are used to treat various disease and ailments in traditional Chinese medicinal and ethnomedicinal practice. Uses include expectorant, curing dyspepsia, sleep problems, asthma, nausea, diarrhea, lumbar-leg pain and arthralgia.[1] The plant mainly contains amide alkaloids which have been used with anti-hepatitis B virus (anti-HBV),[2,3] apoptotic,[4] leishmanicidal,[5] cytotoxic,[6] mosquito larvicidal,[7,8] phytotoxic,[9] anti-inflammatory,[10] antihyper-lipidemic,[11,12] cell adhesion inhibitory,[13] antiplatelet,[11,14] acyl-CoA: cholesterol acyltransferase (ACAT) inhibitory,[15] anti-fungal[16] and coronary vasorelaxant activities.[17] In addition to amide alkaloids, phytochemicals present also include prenylated phenolic compounds[18] and aromatic esters[19] are found in the plant. In our continuing research on bioactive constituents of *Piper* species,[20–22] three new amide alkaloids piperlongumamides A–C (**1**–**3**), along with 12 previously identified ones (Figure 1) were isolated from the fruits of *P. longum*. The structural elucidation of the new compounds and the bioassay results are reported.

*To whom correspondence should be addressed. E-mail: wangyuehu@mail.kib.ac.cn (Y.H. Wang); long@mail.kib.ac.cn (C.L. Long)

## Results and Discussion

The molecular formula of compound **1**, $C_{22}H_{31}NO$, was determined using the HREIMS (*m/z* 325.2413 $[M]^+$), indicating eight degrees of unsaturation. Its IR spectrum showed strong absorption bands at 1722, 1636 and 1452 $cm^{-1}$ implying the existence of unsaturated amide and aromatic functionalities. The $^1H$ NMR and $^{13}C$ NMR spectra of **1** (Table 1) showed signals for one monosubstituted benzene ring [$\delta_H$ 7.27 (2H, m) and 7.17 (3H, m)], two conjugated *trans* double bonds [$\delta_H$ 7.30 (dd, $J$ = 14.8, 10.8 Hz), 6.24 (d, $J$ = 14.8 Hz), 6.17 (dd, $J$ = 15.2, 10.8 Hz) and 6.08 (dt, $J$ = 15.2, 7.0 Hz)], one carbonyl group ($\delta_C$ 166.0) and several methylene groups. Moreover, amide alkaloids are major constituents of *Piper* plants, and the compound might be a phenylalkenoyl derivative. A piperidine ring was determined by the $^1H$-$^1H$ COSY spectrum (Figure 2). The length of the alkenoyl group was determined as 11 carbon atoms according to the molecular formula of compound **1**. Finally, on the basis of the HMBC correlations (Figure 2) from H-3 to C-1, $H_2$-1″ and $H_2$-5″ to C-1, $H_2$-10 to C-1′ and $H_2$-11 to C-2′ and C-6′, the structure of **1** was determined to be (2*E*,4*E*)-*N*-(11-phenylundecadienoyl)piperidine and was given the common name piperlongumamide A.

The molecular formula of compound **2**, $C_{22}H_{33}NO$, was determined by HREIMS at *m/z* 327.2554 $[M]^+$. Its IR spectrum showed absorption bands at 1723, 1657, 1618 and 1439 $cm^{-1}$ indicating the existence of unsaturated amide and aromatic groups. Comparison of the MS and NMR data of **2** with those of **1** (Table 1), the difference was that a *trans* double bond at

**Figure 1.** Structures of amide alkaloids **1**–**15** from *Piper longum*

C-4(5) disappeared in compound **2**. Based on the $^1$H-$^1$H COSY and HMBC correlations of **2** (Figure 2), the structure of **2** was deduced to be (2*E*)-*N*-(11-phenylundecenoyl)piperidine and was given the common name piperlongumamide B.

Compound **3** has the molecular formula $C_{19}H_{31}NO$ based on HREIMS at *m/z* 289.2414 $[M]^+$. The IR spectrum indicated the presence of an unsaturated amide group (1720, 1713, 1630 and 1447 cm$^{-1}$). The $^1$H and $^{13}$C NMR spectra of **3** (Table 2) displayed signals for two *trans*-conjugated diene moiety [$\delta_H$ 6.24 (1H, d, $J$ = 15.0 Hz, H-2), 7.29 (1H, dd, $J$ = 15.0, 11.0 Hz, H-3), 6.18 (1H, dd, $J$ = 15.0, 11.0 Hz, H-4) and 6.08 (1H, dt, $J$ = 15.0, 7.2 Hz, H-5)], one methyl group [$\delta_H$ 0.89 (3H, t, $J$ = 6.8 Hz, H-14)], one carbonyl group ($\delta_H$ 166.1) and several methylene groups. Also, signals at $\delta_H$ 5.32 (1H, m, H-9) and 5.38 (1H, m, H-10) were due to an additional double bond, which was deduced as *Z* configuration by the chemical shifts of the allylic carbons [$\delta_C$ 26.8 (C-8) and 27.1 (C-11)].[21,22] Based on above analysis and by comparison of its NMR data with those of compound **1**, compound **3** was deduced as an alkenoyl piperidine. According to $^1$H-$^1$H COSY correlations of **3** (Figure 2), the connections from C-1 to C-11, C-13 to C-14 and C-1′ to C-5′ were confirmed. Finally, based on the HMBC

**Figure 2.** Key $^1$H-$^1$H COSY (bold) and HMBC (arrows, H→C) correlations of compounds **1**–**3**

**Table 1. $^1$H and $^{13}$C NMR spectral data of 1 and 2 in $CDCl_3$ ($\delta$ in ppm)**

| No. | 1 | | 2 | |
|---|---|---|---|---|
| | $\delta_C$ (150 MHz) | $\delta_H$ (600 MHz) | $\delta_C$ (100 MHz) | $\delta_H$ (400 MHz) |
| 1 | 166.0 C | | 165.8 C | |
| 2 | 118.0 CH | 6.24, 1H, d (14.8) | 120.2 CH | 6.23, 1H, br. d (15.1) |
| 3 | 143.7 CH | 7.30,1H, dd (14.8, 10.8) | 146.6 CH | 6.84,1H, dt (15.1, 7.0) |
| 4 | 128.9 CH | 6.17, 1H, dd (15.2, 10.8) | 32.7 $CH_2$ | 2.18, 2H, q like (7.0) |
| 5 | 143.3 CH | 6.08, 1H, dt (15.2, 7.0) | 28.5 $CH_2$ | 1.43, 2H, m |
| 6 | 33.1 $CH_2$ | 2.14, 2H, q like (7.0) | 29.5[b] $CH_2$ | 1.29, 2H, m |
| 7 | 28.8 $CH_2$ | 1.41, 2H, m | 29.5[b] $CH_2$ | 1.29, 2H, m |
| 8 | 29.2 $CH_2$ | 1.32, 2H, m | 29.4[b] $CH_2$ | 1.29, 2H, m |
| 9 | 29.2 $CH_2$ | 1.32, 2H, m | 29.3[b] $CH_2$ | 1.29, 2H, m |
| 10 | 31.6 $CH_2$ | 1.60, 2H, m | 31.6 $CH_2$ | 1.60, 2H, m |
| 11 | 36.0 $CH_2$ | 2.59, 2H, t (7.8) | 36.1 $CH_2$ | 2.59, 2H, t (7.7) |
| 1′ | 142.9 C | | 143.0 C | |
| 2′,6′ | 128.4 CH | 7.27, 2H, m | 128.3 CH | 7.27, 2H, m |
| 3′,5′ | 128.5 CH | 7.17, 2H, m | 128.5 CH | 7.18, 2H, m |
| 4′ | 125.7 CH | 7.17, 1H, m | 125.7 CH | 7.18, 1H, m |
| 1″ | 45.2[a] $CH_2$ | 3.57, 2H, m | 42.9[a] $CH_2$ | 3.54, 2H, m |
| 2″ | 26.3 $CH_2$ | 1.58, 2H, m | 26.2 $CH_2$ | 1.56, 2H, m |
| 3″ | 24.7 $CH_2$ | 1.65, 2H, m | 24.8 $CH_2$ | 1.65, 2H, m |
| 4″ | 26.3 $CH_2$ | 1.58, 2H, m | 26.2 $CH_2$ | 1.56, 2H, m |
| 5″ | 45.2[a] $CH_2$ | 3.56, 2H, m | 46.9[a] $CH_2$ | 3.54, 2H, m |

[a]Detected by HSQC spectrum. [b]Data under the same entry are exchangeable.

correlations (Figure 2) from H-3, $H_2$-1′ and $H_2$-5′ to C-1, H-10 to C-12, Me-14 to C-12 and C-13, the structure of **3** was determined to be (2*E*,4*E*,9*Z*)-*N*-tetradecatrienoylpiperidine and was given the common name piperlongumamide C.

The known compounds were identified as piperine (**4**),[23] isopiperine (**5**),[24] chavicine (**6**),[24] piperlonguminine (**7**),[25] pellitorine (**8**),[26] brachystamide B (**9**),[27] guineensine (**10**),[25] piperchabamide B (**11**),[28] (2*E*,4*E*)-*N*-dodecadienoylpiperidine (**12**),[29] dehydropipernonaline (**13**),[12] pipernonaline (**14**)[16] and piperolein B (**15**)[12] by comparison of their NMR and MS data with those reported in the literature.

In a previous study, we found that an amide alkaloid 1-[(9*E*)-10-(3,4-methylenedioxyphenyl)-9-decenoyl]pyrrolidine from *P. boehmeriaefolium* was cytotoxic.[21] Therefore, all of the amides from *P. longum* were evaluated for their inhibitory activities against HL-60 (human leukemia), A-549 (human lung cancer), MCF-7 (human breast cancer), SMMC-7721 (human liver cancer) and SW480 (human rectal cancer) cell lines. Piperchabamide B (**11**) exhibited weak inhibitory activity against HL-60 cell line ($IC_{50}$ = 21.32 μM), A-549 ($IC_{50}$ = 23.82 μM) and MCF-7 ($IC_{50}$ = 16.58 μM) (Table 3). Other tested compounds were inactive.

**Table 2. $^1$H (600 MHz) and $^{13}$C NMR (150 MHz) spectral data of 3 in $CDCl_3$ ($\delta$ in ppm)**

| No. | $\delta_C$ | $\delta_H$ |
|---|---|---|
| 1 | 166.1 C | |
| 2 | 117.8 CH | 6.24, 1H, d (15.0) |
| 3 | 144.1 CH | 7.29, 1H, dd (15.0, 11.0) |
| 4 | 129.1 CH | 6.18, 1H, dd (15.0, 11.0) |
| 5 | 143.4 CH | 6.08, 1H, dt (15.0, 7.2) |
| 6 | 32.6 $CH_2$ | 2.16, 2H, q like (7.2) |
| 7 | 28.9 $CH_2$ | 1.48, 2H, m |
| 8 | 26.8 $CH_2$ | 2.04, 2H, m |
| 9 | 129.1 CH | 5.32, 1H, m |
| 10 | 130.7 CH | 5.38, 1H, m |
| 11 | 27.1 $CH_2$ | 2.01, 2H, m |
| 12 | 32.0 $CH_2$ | 1.31, 2H, m |
| 13 | 22.5 $CH_2$ | 1.31, 2H, m |
| 14 | 14.2 $CH_3$ | 0.89, 3H, t (6.8) |
| 1′ | 45.2[a] $CH_2$ | 3.56, 2H, m |
| 2′ | 26.3 $CH_2$ | 1.58, 2H, m |
| 3′ | 24.7 $CH_2$ | 1.66, 2H, m |
| 4′ | 26.3 $CH_2$ | 1.58, 2H, m |
| 5′ | 45.2[a] $CH_2$ | 3.56, 2H, m |

[a]Detected by HSQC spectrum.

## Experimental Section

**General Experimental Procedures.** UV spectra were recorded on a Shimadzu double-beam 210A spectrometer (Shimadzu Co., Shimadzu, Japan). IR spectra were recorded on a Bruker Tensor 27 Fourier transform infrared spectrometer (Bruker, Karlsruhe, Germany) with KBr pellets. ESIMS and HREIMS analyses were carried out on an API Qstar-Pulsar-1 mass spectrometer (Applied Biosystems/MDS Sciex, Ontario, Canada) and Waters AutoSpec Premier P776 (Waters, Milford, USA), respectively. $^1$H and $^{13}$C NMR spectra were collected on a Bruker AM-400, DRX-500 and Avance III-600 spectrometers (Bruker Bio-Spin GmbH, Rheinstetten, Germany) with TMS as an internal standard. Semi-preparative HPLC was performed on an Agilent 1200 series pump (Agilent Technologies, Santa Clara, USA) equipped with a diode array detector and a Zorbax SB-$C_{18}$ column (5.0 μm, $\phi$ 9.4 × 250 mm). Both analytical and preparative TLC conductedusing silica gel plates ($GF_{254}$, Yantai Institute of Chemical Technology, Yantai, China). The spots were initially visualized using UV light (254 and 366 nm) and subsequently visualised by spraying a solution of 5% $H_2SO_4$ onto the TLC plate, which was subsequently heated. Column chromatography was performed using silica gel (80–100 mesh and 300–400 mesh; Qingdao Makall Group Co., Ltd., Qingdao, China), $C_{18}$ silica gel (40–75 μm, Fuji Silysia Chemical, Ltd., Kasugai, Japan) and Sephadex LH-20 (GE Healthcare Bio-Xciences AB, Uppsala, Sweden).

**Plant Material.** The fruits of *P. longum* were purchased from Yikan Chinese Herbal Medicine Ltd., Qujing, China, in October 2011. The plant material was identified by Dr. Guang-Wan Hu, at Kunming Institute of Botany, Chinese Academy of Sciences. A voucher specimen (QJ1101) has been deposited at Key Laboratory of Economic Plants and Biotechnology, Kunming Institute of Botany.

**Table 3. Cytotoxicity of piperchabamide B (11) from *Piper longum****

| Compound | $IC_{50}$ (μM) | | | | |
|---|---|---|---|---|---|
| | HL-60 | SMMC-7721 | A-549 | MCF-7 | SW480 |
| Piperchabamide B (**11**) | 21.32 | > 40 | 23.82 | 16.58 | > 40 |
| Cisplatin (positive control) | 1.32 | 6.24 | 11.83 | 15.17 | 12.95 |
| Taxol (positive control) | < 0.008 | < 0.008 | < 0.008 | < 0.008 | < 0.008 |

*Other tested compounds were inactive.

**Extraction and Isolation.** The dried powdered fruits (20 kg) of *P. longum* were extracted using MeOH (4, 3 and 3 h, resp.) under reflux. The combined MeOH extracts were evaporated under reduced pressure to yield a residue, which was suspended in $H_2O$ and then partitioned successively with petroleum ether and $CHCl_3$ to produce two corresponding portions. After TLC testing, the two portions were combined as both contained alkaloids. The combined extract (1377 g) was subjected to column chromatography over silica gel G (80–100 mesh) using petroleum ether/EtOAc (1:0→0:1) to yield ten fractions (Fr. A–J) and also compound **4** (1.8 g).

Fr. D (19 g) was separated using column chromatography (C18, MeOH/$H_2O$, 80:20) to give **8** (56.2 mg). Fr. E (12 g) was partitioned by column chromatography (C18, MeOH/$H_2O$, 60:40→95:5) to produce fractions (E1–E7). Fr. E1 (1.1 g) was fractionated by column chromatography (Sephadex LH-20, MeOH; silica gel, petroleum ether/$Me_2CO$, 1:1) and prep. TLC (petroleum ether/EtOAc, 10:1) to give **5** (20.0 mg), **6** (27.0 mg) and **7** (8.2 mg).

Fr. E3 (2.5 g) was fractionated by column chromatography (Sephadex LH-20, MeOH; silica gel, petroleum ether/$Me_2CO$, 3:1) and semi-preparative HPLC (MeOH/$Me_3CN$/$H_2O$, 50:35:15, 4 mL/min) to obtain **13** (23.8 mg, $t_R$ = 5.995 min) and **14** (37.3 mg, $t_R$ = 6.818 min). Fr. E4 was fractionated by column chromatography (Sephadex LH-20, MeOH) to give two parts (E4a and E4b). Fr. E4a (233.6 mg) was subjected to by column chromatography (silica gel, petroleum ether/$Et_2NH$, 50:1) and semi-preparative HPLC (MeCN/$H_2O$, 90:10, 3 ml/min) to give **12** (1 mg, $t_R$ = 11.984 min), **1** (2.1 mg, $t_R$ = 12.858 min), **2** (4 mg, $t_R$ = 16.182 min), **3** (8.4 mg, $t_R$ = 13.959 min). Fr. E4b (743.7 mg) was isolated repeatedly by column chromatography (silica gel, petroleum ether/$Me_2CO$, 5:1; petroleum ether/EtOAc, 4:1; petroleum ether/$Et_2NH$, 10:1) to give **10** (17.2 mg) and **11** (18.0 mg), and a remaining fraction, which was purified by semi-preparative HPLC (MeCN/$H_2O$, 60:40, 4 mL/min) to give **15** (3.5 mg, $t_R$ = 17.027 min) and **11** (4 mg, $t_R$ = 28.529 min). Fr. E5 (1.3 g) was fractionated by column chromatography (Sephadex LH-20, MeOH) and prep. TLC ($CHCl_3$/EtOAc, 10:1) to give **9** (23.9 mg).

**Piperlongumamide A (1):** pale yellow oil; UV (MeOH): $\lambda_{max}$ (log ε) 205 (2.77) nm. IR (KBr) $\nu_{max}$ 1722, 1636, 1452, 1254, 748, 699, 571 $cm^{-1}$. $^1H$ and $^{13}C$ NMR spectral data see Table 1. ESIMS (positive) *m/z* 326 $[M + H]^+$; HREIMS: *m/z* 325.2413 $[M]^+$ (calcd for $C_{22}H_{31}NO$, 325.2406).

**Piperlongumamide B (2):** pale yellow oil; UV (MeOH): $\lambda_{max}$ (log ε) 209 (2.93) nm. IR (KBr) $\nu_{max}$ 1723, 1657, 1618, 1439, 1219, 1069, 1023, 974, 903, 637 $cm^{-1}$. $^1H$ and $^{13}C$ NMR spectral data see Table 1. ESIMS (positive) *m/z* 350 $[M + Na]^+$; HREIMS: *m/z* 327.2554 $[M]^+$ (calcd for $C_{22}H_{33}NO$, 327.2562).

**Piperlongumamide C (3):** pale yellow oil; UV (MeOH): $\lambda_{max}$ (log ε) 207 (2.33) nm. IR (KBr) $\nu_{max}$ 1720, 1713, 1630, 1447, 1253, 1229, 1166, 1082, 1022, 978, 800, 588 $cm^{-1}$. $^1H$ and $^{13}C$ NMR spectral data see Table 2. ESIMS (positive) *m/z* 290 $[M + H]^+$; HREIMS: *m/z* 289.2414 $[M]^+$ (calcd for $C_{19}H_{31}NO$, 289.2406).

**MTS Assay for Cytotoxicity.** The isolated amide alkaloids were tested *in vitro* for their cytotoxicity against proliferation of human leukemia HL-60 cell line, human lung cancer A-549, human breast cancer MCF-7, human liver cancer SMMC-7721 and human rectal cancer SW480 using the MTS assay.

The 3-(4,5-dimethylthiazol-2-yl)-5(3-carboxymethoxy-phenyl)-2-(4-sulfopheny)-2*H*-tetrazolium (MTS) (Promega, Beijing, China) is a mix-based cell titer assay and was performed as previous described.[30] Initially, cells in their log-phase of their cycle were seeded in 96-well plates (5000–10000 cells/well, NEST Biotechnology, Wuxi, China) using a standard 100 μL volume, with paired single cell suspension containing 10% fetal bovine serum (DMEM or RMPI1640, Thermo Fisher Scientific, Beijing, China). The cell were further treated with indicated concentrations of the compounds dissolved in DMSO, which were set the regular thickness 40 μM for the initial screening and five concentrations of each compound were fixed in the concentration compounds inhibited tumor cell growth by approximately 50% to achieve a total culture medium in a volume of 200 μL. After incubation for 48 h at 37 °C, a 20 μL of MTS solution and 100 μL DMEM were added into the well, incubation was continued for another 1–4 h. The absorbance was measured at the detection wavelength of 490 nm ($L_1$) and the reference wavelength of 680 nm ($L_2$)[31] and cytotoxicity for each compound was expressed as $IC_{50}$ values.

## Electronic Supplementary Material

Supplementary material is available in the online version of this article at http://dx.doi.org/10.1007/s13659-013-0073-0 and is accessible for authorized users.

## Acknowledgments

This work was funded by the Natural Science Foundation of Yunnan Province, China (No. 2011FZ205), the National Natural Science Foundation of China (Nos. 31070288, 31161140345), the Ministry of Education of China through its 111 and 985 projects (Nos. B08044, MUC98506-01000101 & MUC985-9), and the Japan Society for the Promotion of Science (No. JSPS/AP/109080).

**Open Access** This article is distributed under the terms of the Creative Commons Attribution License which permits any use, distribution, and reproduction in any medium, provided the original author(s) and source are credited.

## Electronic supplementary material

Supplementary material, approximately 1.95 MB.
