# Supplementary material for: New amide alkaloids from Piper longum fruits
Source: Nat Prod Bioprospect. 2013 Nov 27;3(6):277–81. doi: 10.1007/s13659-013-0073-0 (PMC4131601; doi:10.1007/s13659-013-0073-0)
Supplement: Supplementary file 1 — Supplementary material, approximately 1.95 MB. [file 13659_2013_73_MOESM1_ESM.pdf]

Electronic Supplementary Material

New amide alkaloids from *Piper longum* fruits

Jun YANG,[a] Yao SU,[a,b] Ji-Feng LUO,[a] Wei GU,[a] Hong-Mei NIU,[a] Yan LI,[c] Yue-Hu WANG,[a,]* and Chun-Lin LONG[a,d,]*

[a]Key Laboratory of Economic Plants and Biotechnology, Kunming Institute of Botany, Chinese Academy of Sciences, Kunming 650201, China

[b]College of Resources and Environmental Sciences, Hebei Agricultural University, Hebei 071000, China

[c]State Key Laboratory of Phytochemisty and Plant Resources in West China, Kunming Institute of Botany, Chinese Academy of Sciences, Kunming 650201, China

[d]College of Life and Environmental Sciences, Minzu University of China, Beijing 100081, China

Received 13 September 2013; Accepted 16 November 2013

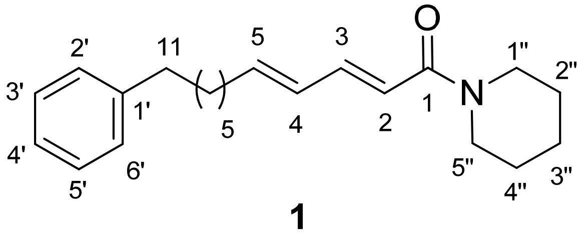

1

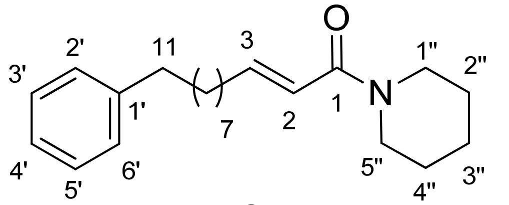

2

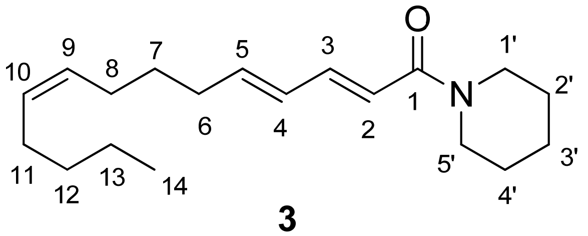

3

Structures of compounds **1**–**3**

*To whom correspondence should be addressed. E-mail: wangyuehu@mail.kib.ac.cn (Y.H. Wang); long@mail.kib.ac.cn (C.L. Long)

Contents

S1 ^{1}H NMR spectrum of **1**	S11 HSQC spectrum of **2**	S21 ^{1}H – ^{1}H COSY spectrum of **3**
S2 ^{13}C NMR spectrum of **1**	S12 HMBC spectrum of **2**	S22 HREIMS spectrum of **3**
S3 HSQC spectrum of **1**	S13 ^{1}H – ^{1}H COSY spectrum of **2**	S23 ESI-MS spectrum of **3**
S4 HMBC spectrum of **1**	S14 HREIMS spectrum of **2**	S24 UV spectrum of **3**
S5 ^{1}H – ^{1}H COSY spectrum of **1**	S15 ESI-MS spectrum of **2**	
S6 HREIMS spectrum of **1**	S16 UV spectrum of **2**	
S7 ESI-MS spectrum of **1**	S17 ^{1}H NMR spectrum of **3**	
S8 UV spectrum of **1**	S18 ^{13}C NMR spectrum of **3**	
S9 ^{1}H NMR spectrum of **2**	S19 HSQC spectrum of **3**	
S10 ^{13}C NMR spectrum of **2**	S20 HMBC spectrum of **3**	

S1 ^{1}H NMR ($CDCl_3$, 600 MHz) spectrum of 1

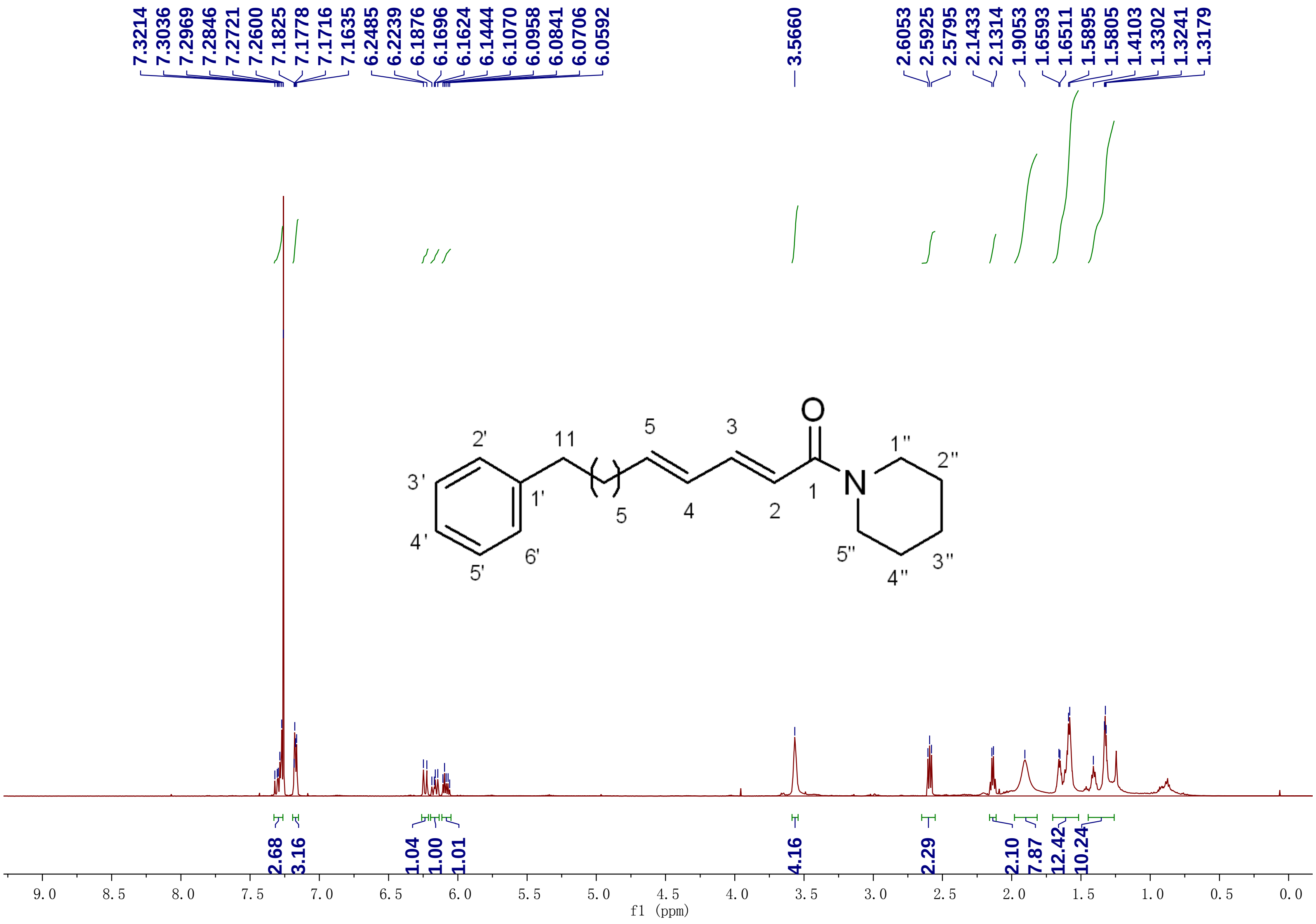

S2 ^{13}C NMR ($CDCl_3$, 150 MHz) spectrum of 1

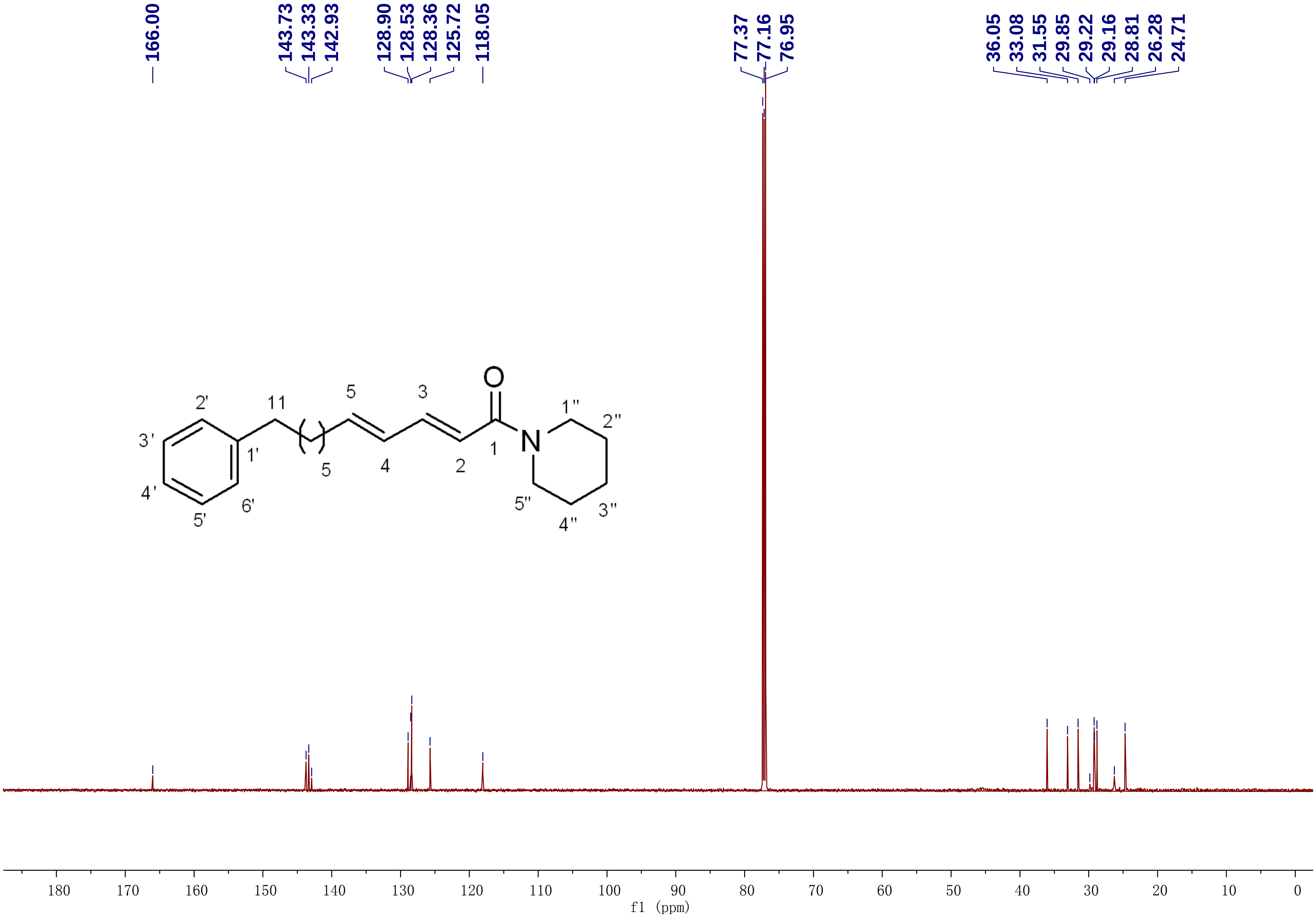

S3 HSQC spectrum of 1

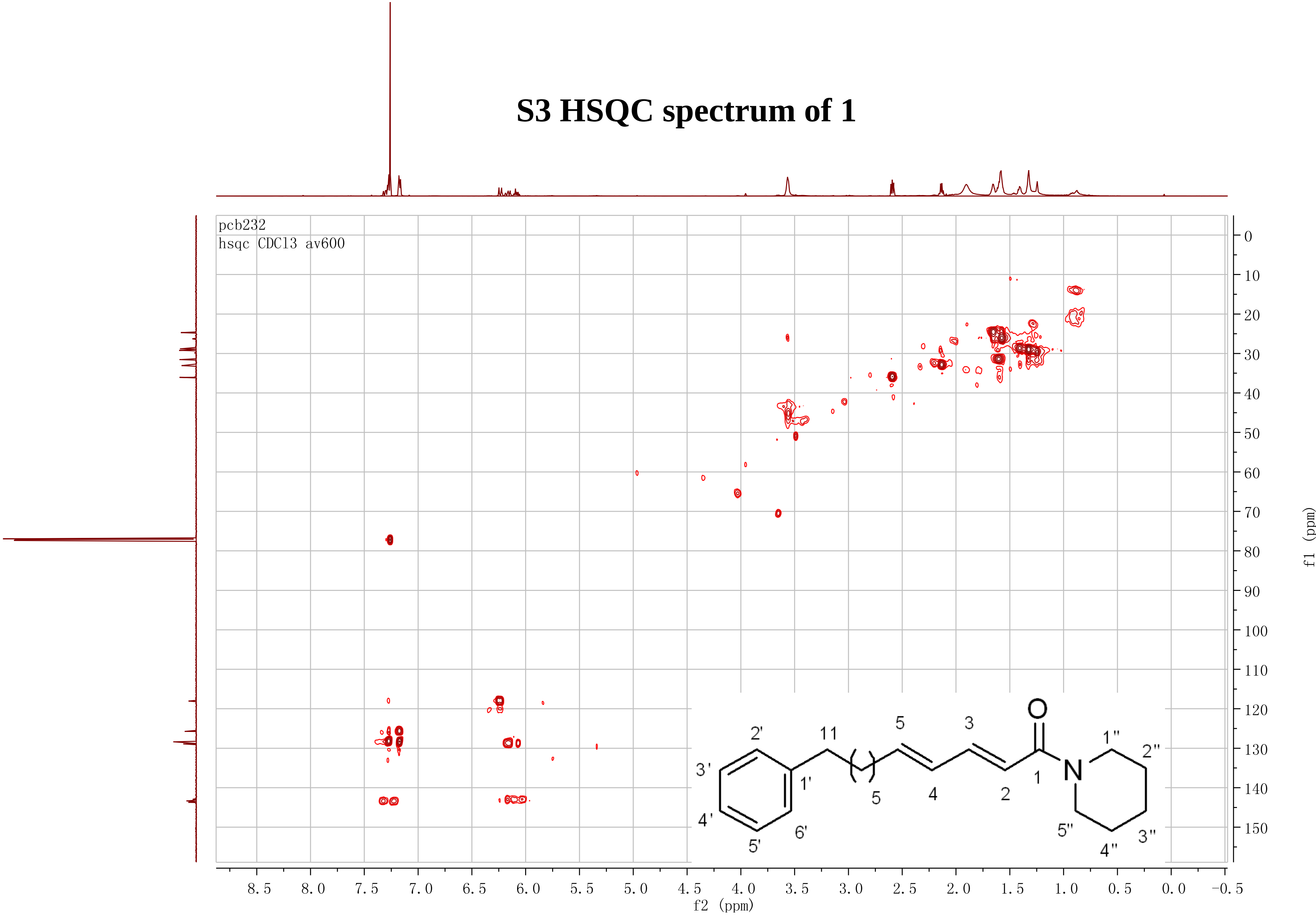

S4 HMBC spectrum of 1

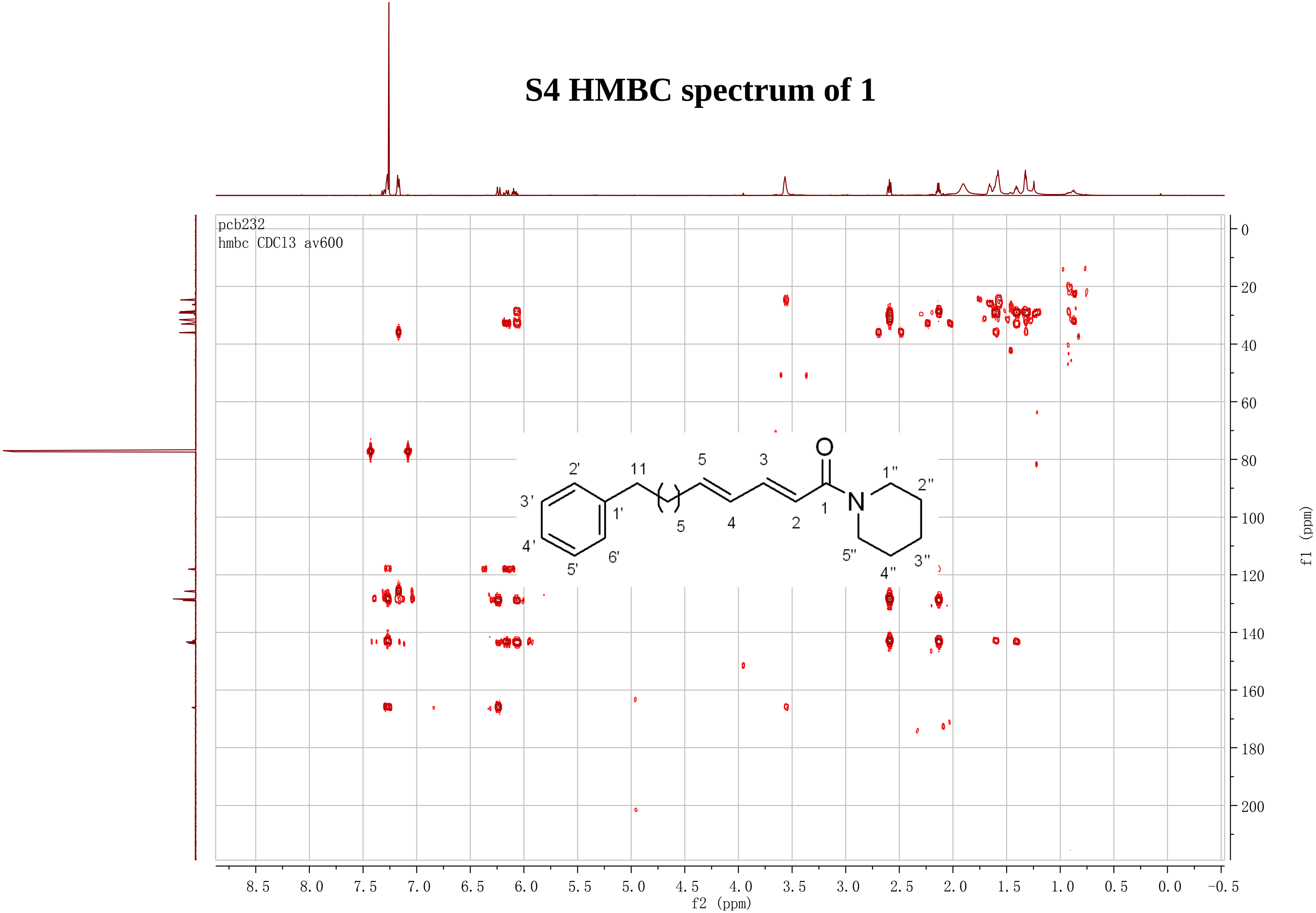

S5 ^{1}H-^{1}H COSY spectrum of 1

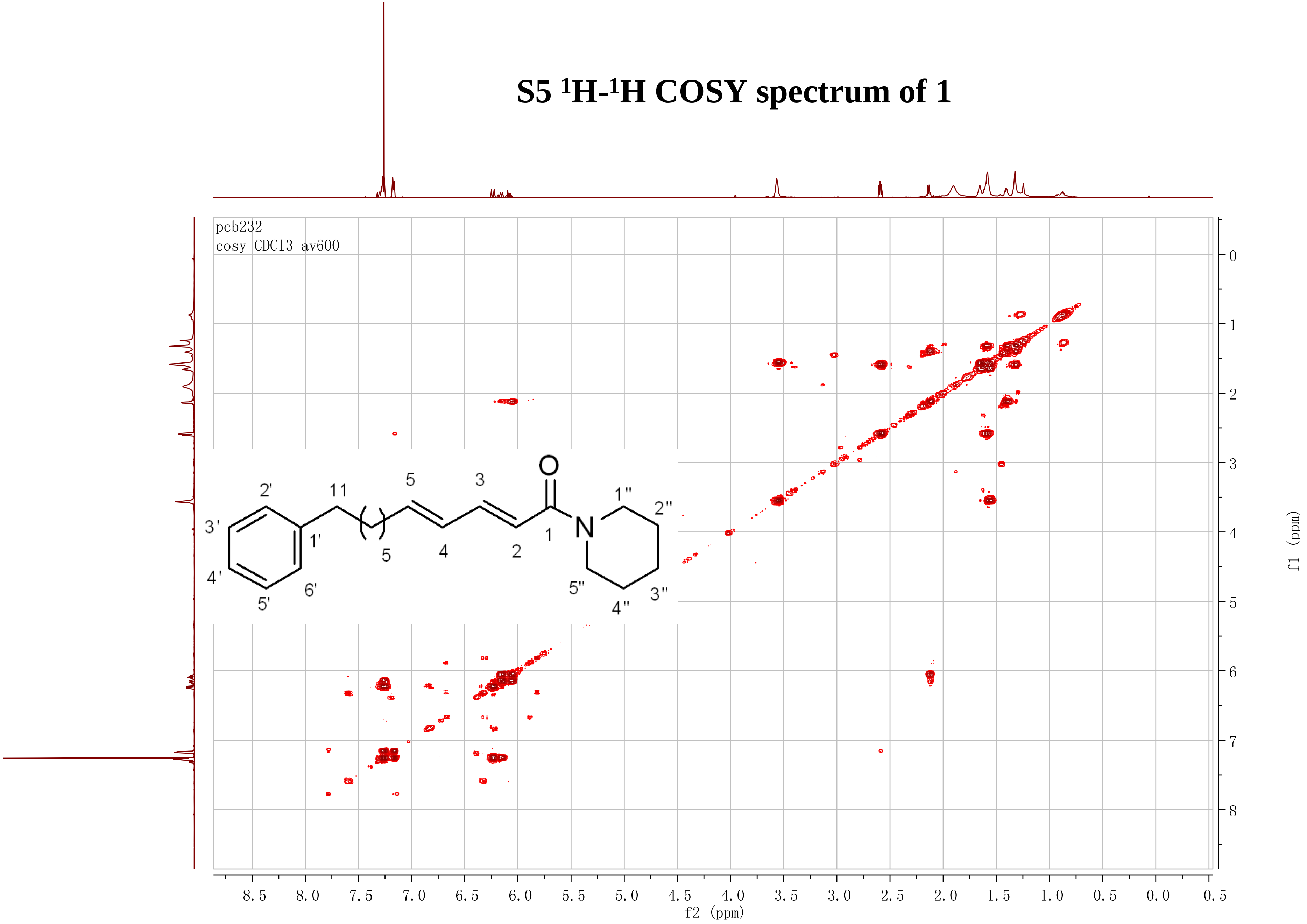

S6 HREIMS spectrum of 1

Single Mass Analysis

Tolerance = 10.0 PPM / DBE: min = -10.0, max = 120.0

Selected filters: None

Monoisotopic Mass, Odd and Even Electron Ions

18 formula(e) evaluated with 1 results within limits (up to 51 closest results for each mass)

Elements Used:

C: 0-200 H: 0-400 N: 1-1 O: 0-2

pcb232 KIB Autospec Premier

16:18:55 12-Nov-2012 M121112EA-16AFAMM 16 (1.469) P776

Voltage EI+ 199

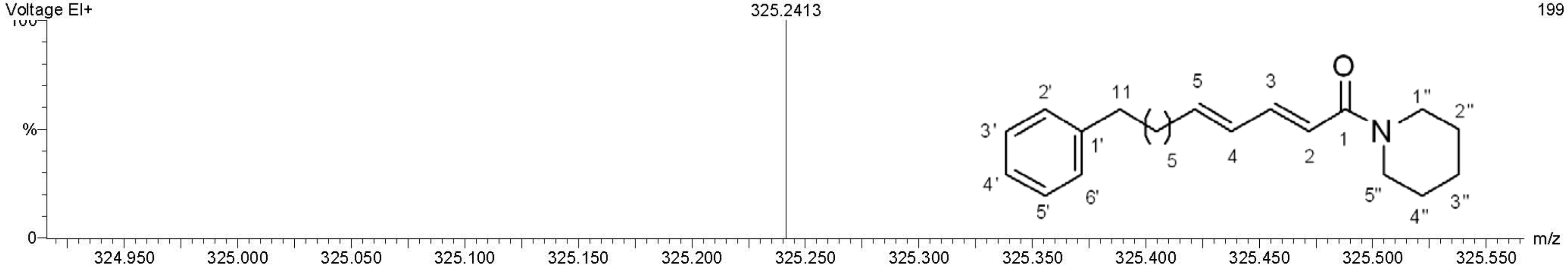

Minimum:				-10.0		
Maximum:		100.0	10.0	120.0		
Mass	Calc. Mass	mDa	PPM	DBE	i-FIT	Formula
325.2413	325.2406	0.7	2.2	8.0	5546114.0	C22 H31 N O

Display Report

**Analysis Info**		Acquisition Date	10/24/2012 4:26:39 PM
Analysis Name	D:\DATA\2012file\1210\121024\pcb23-21.d		
Method	ms_ptservice.m	Operator	Bruker
Sample Name	pcb23-2	Instrument	HCT
Comment			

Acquisition Parameter

Ion Source Type	ESI	Ion Polarity	Positive	Alternating Ion Polarity	off
Mass Range Mode	Ultra Scan	Scan Begin	100 m/z	Scan End	1000 m/z
Capillary Exit	10.0 Volt	Skimmer	40.0 Volt	Trap Drive	34.4
Accumulation Time	100000 µs	Averages	5 Spectra	Auto MS/MS	off

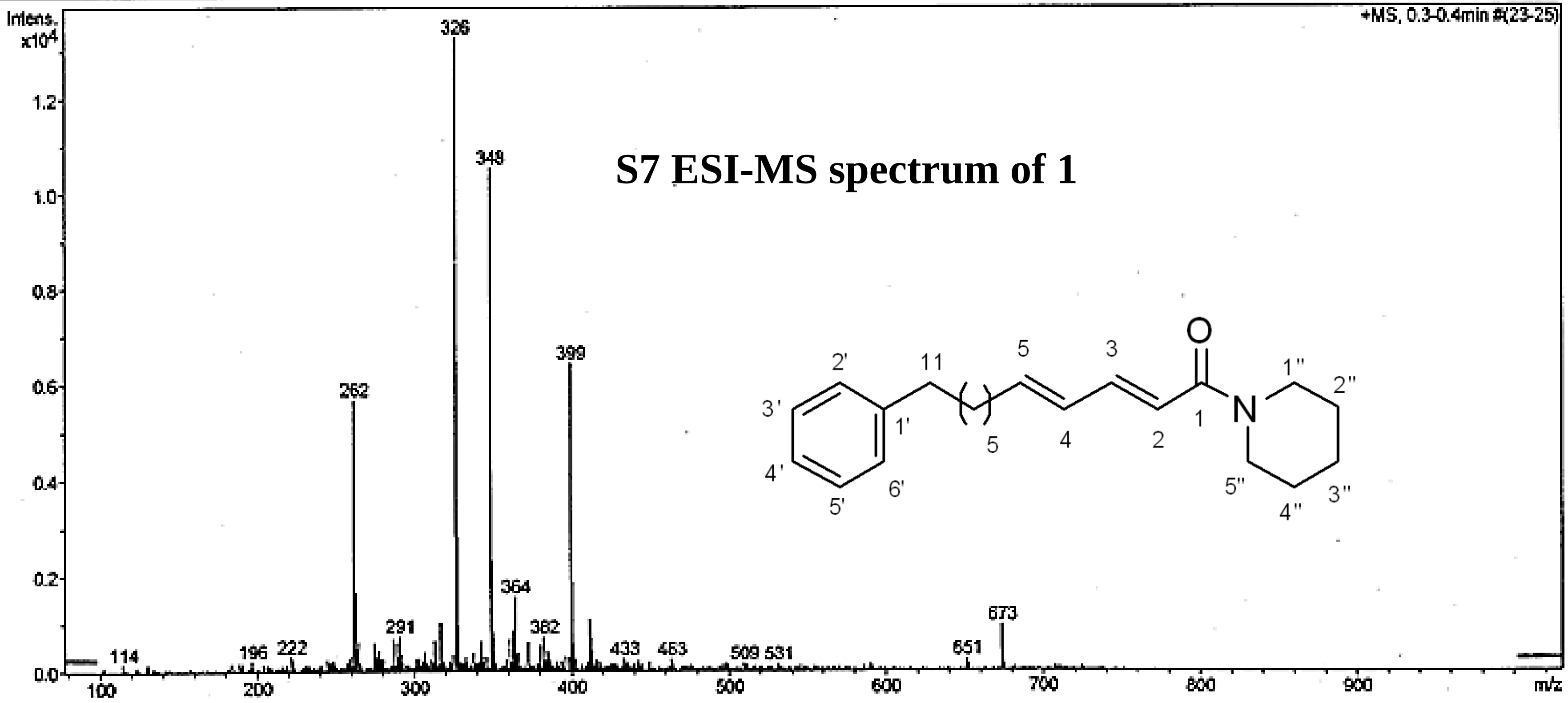

S7 ESI-MS spectrum of 1

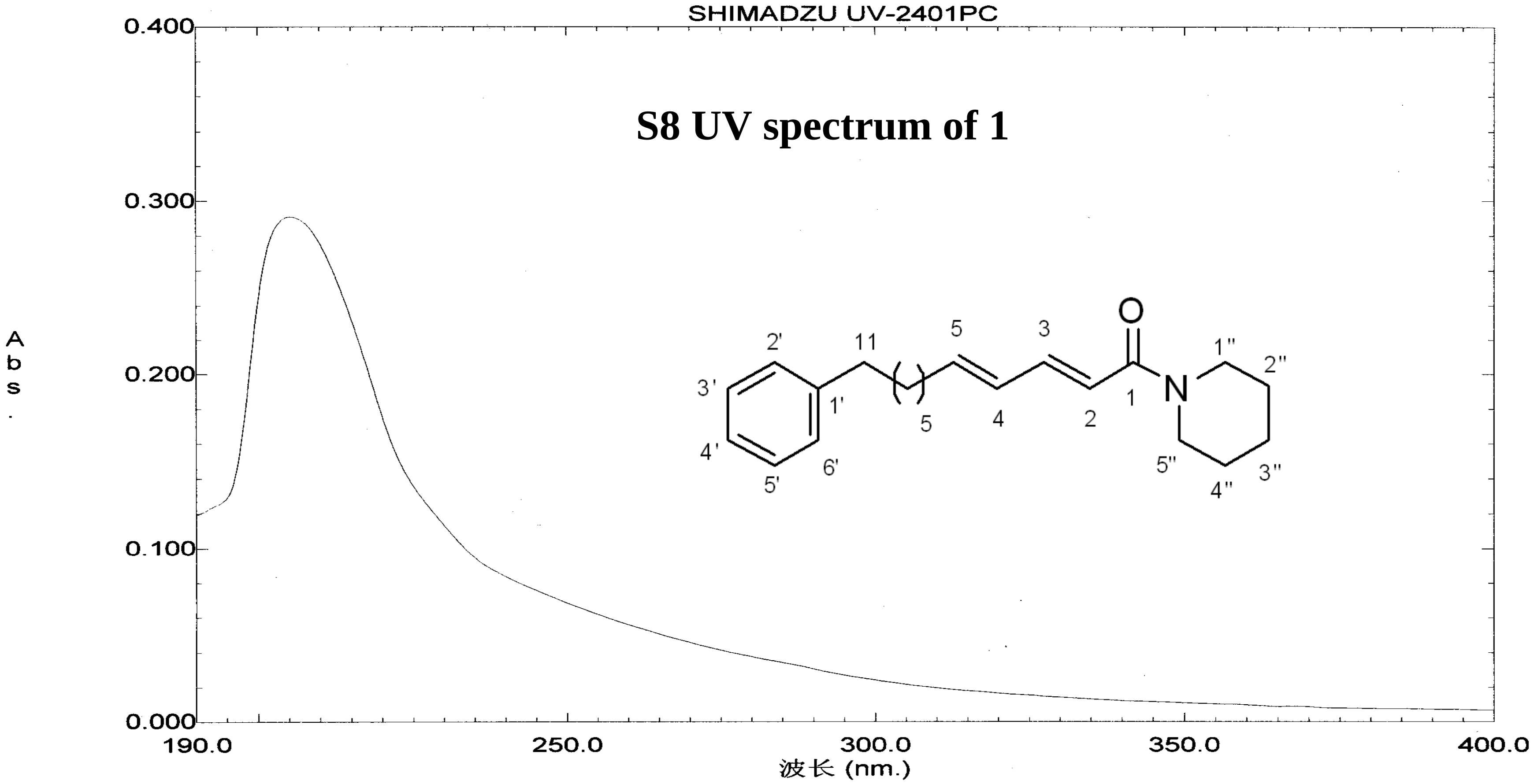

S8 UV spectrum of 1

文件名:　13100904
样品名称：PCB232

创建于:　15:57　13-10-09
数据:　原始

测量模式:　Abs.
扫描速度:　中速
狭缝:　5.0
采样间隔:　0.2

否.	波长 (nm.)	Abs.
1	205.40	0.2908

13100904

样品浓度：0.0320毫克/毫升
溶剂：甲醇

S9 ^1H NMR ($CDCl_3$, 400 MHz) spectrum of 2

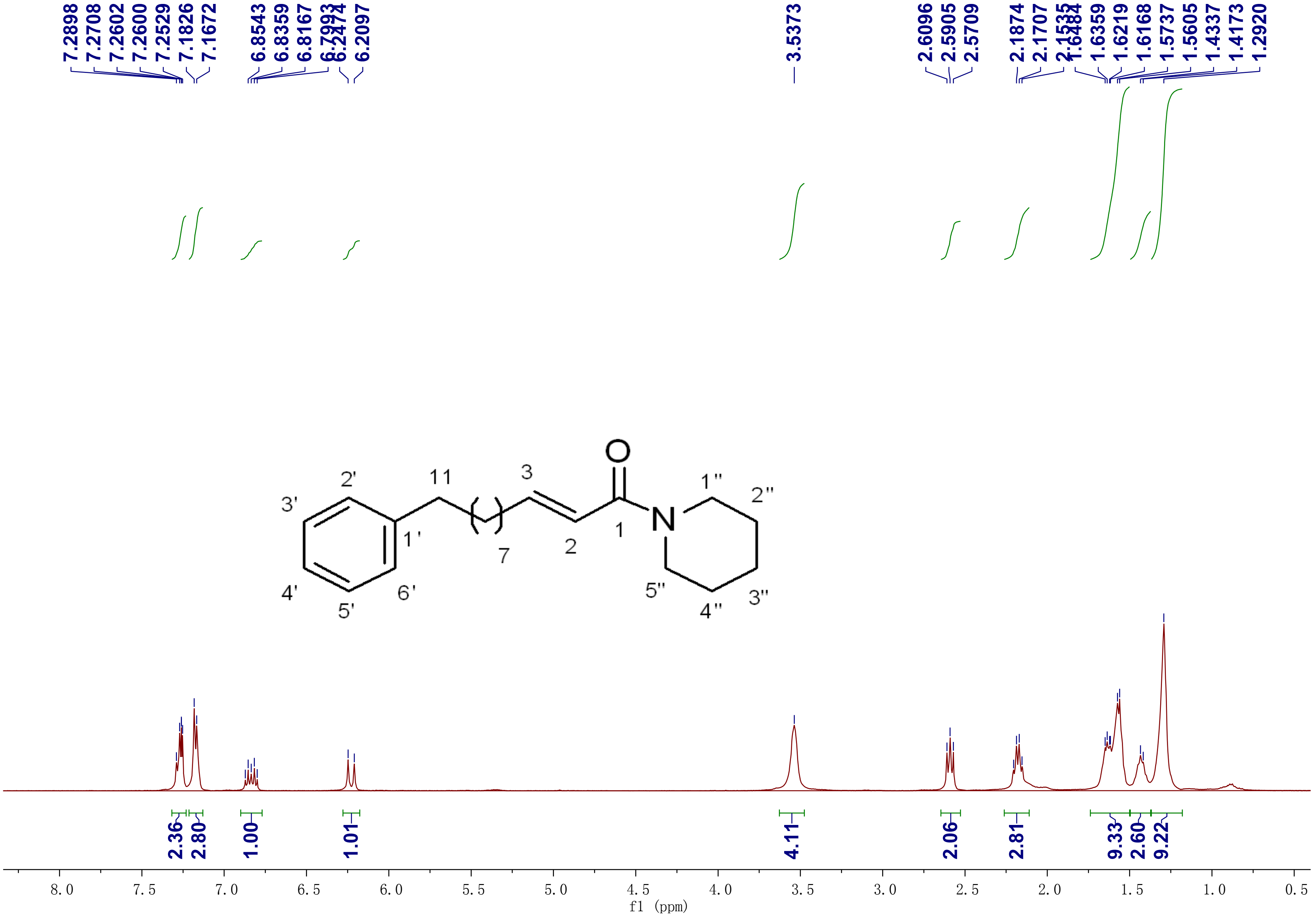

S10 ^{13}C NMR ($CDCl_3$, 100 MHz) spectrum of 2

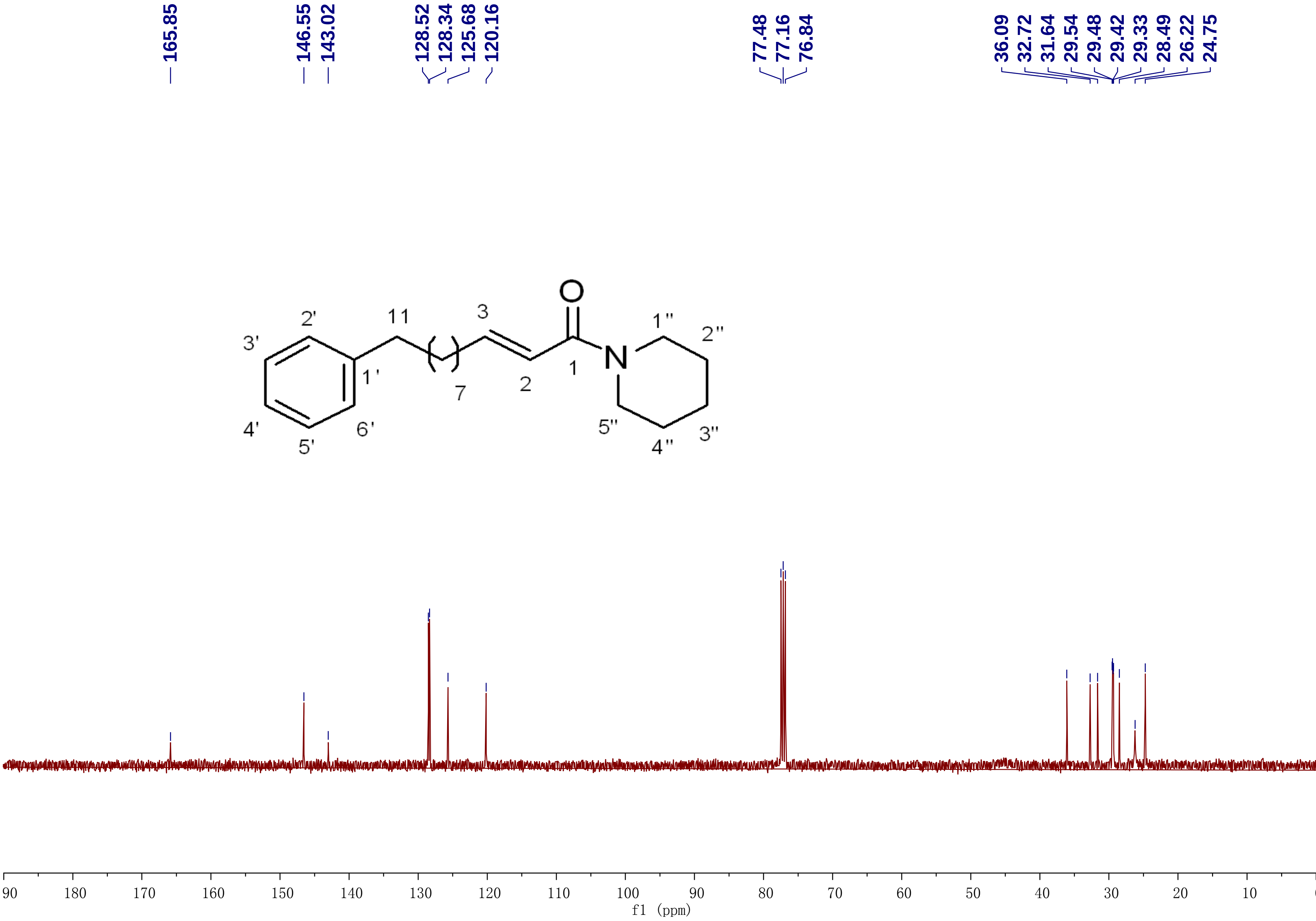

S11 HSQC spectrum of 2

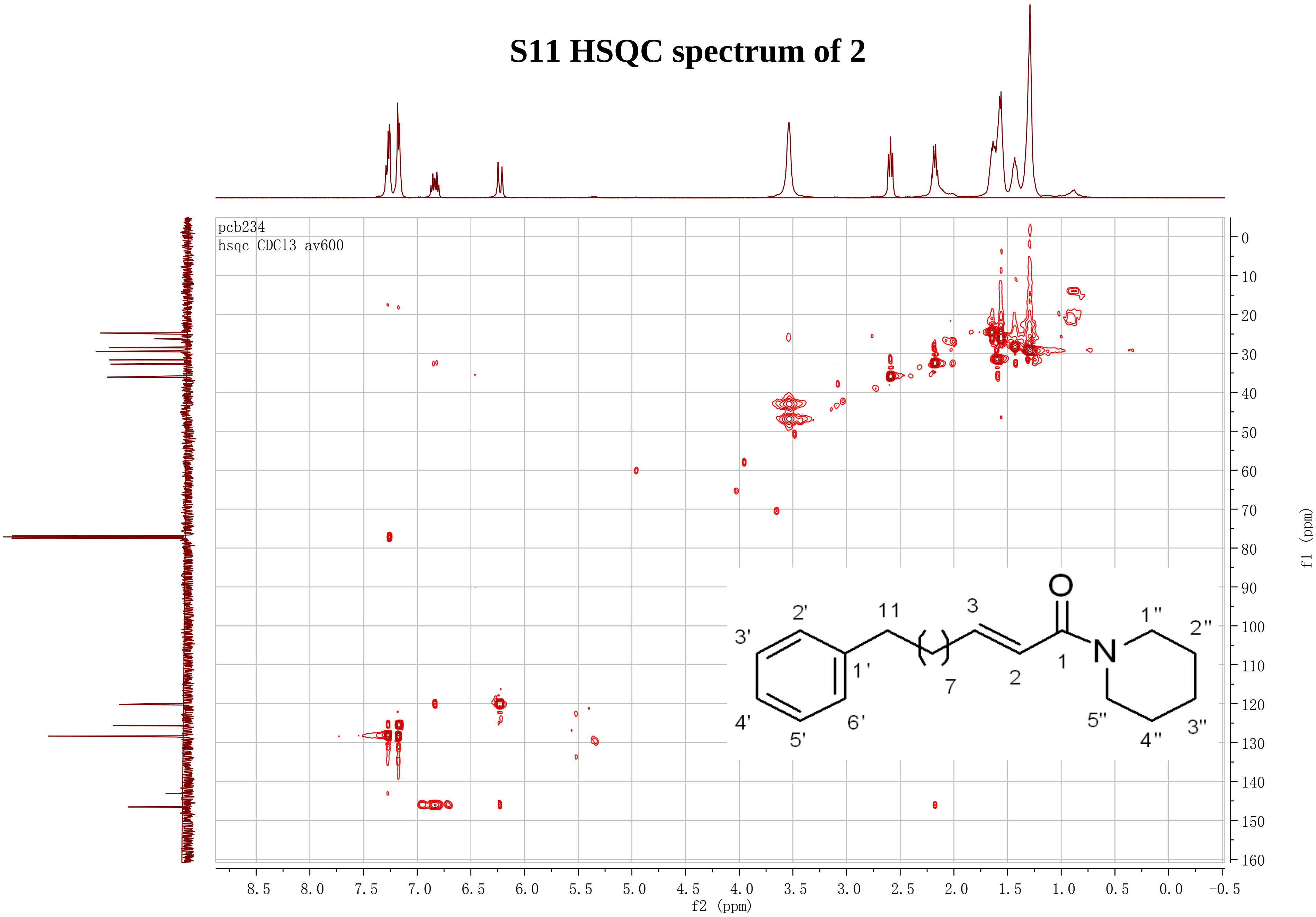

S12 HMBC spectrum of 2

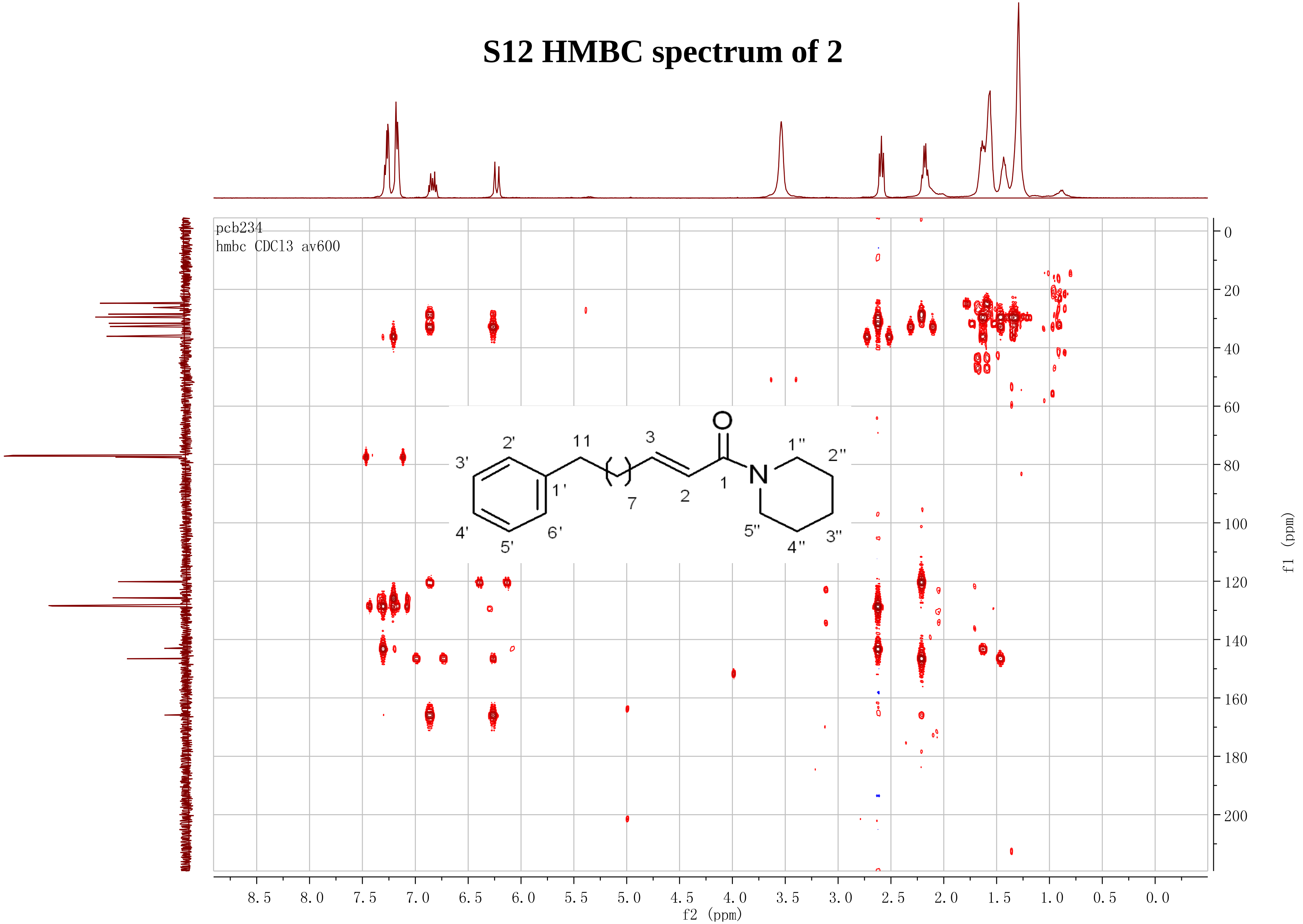

S13 ^1H-^1H COSY spectrum of 2

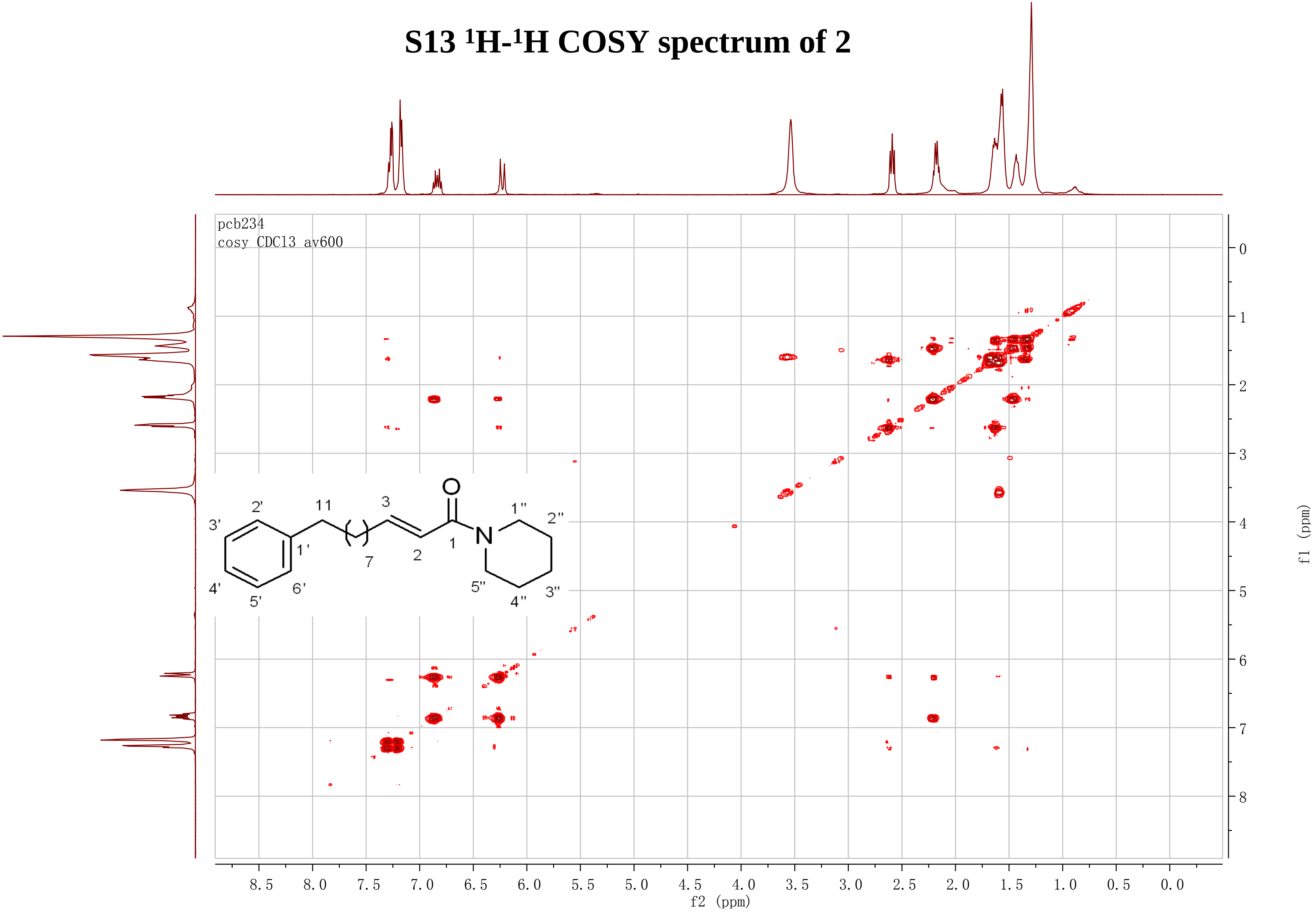

Single Mass Analysis
Tolerance = 10.0 PPM / DBE: min = -10.0, max = 120.0
Selected filters: None

S14 HREIMS spectrum of 2

Monoisotopic Mass, Odd and Even Electron Ions
17 formula(e) evaluated with 1 results within limits (up to 51 closest results for each mass)
Elements Used:
C: 0-200 H: 0-400 N: 1-1 O: 0-2

pcb234
15:50:43 12-Nov-2012
Voltage EI+

KIB
M121112EA-12AFAMM 49 (4.499)

Autospec Premier
P776
560

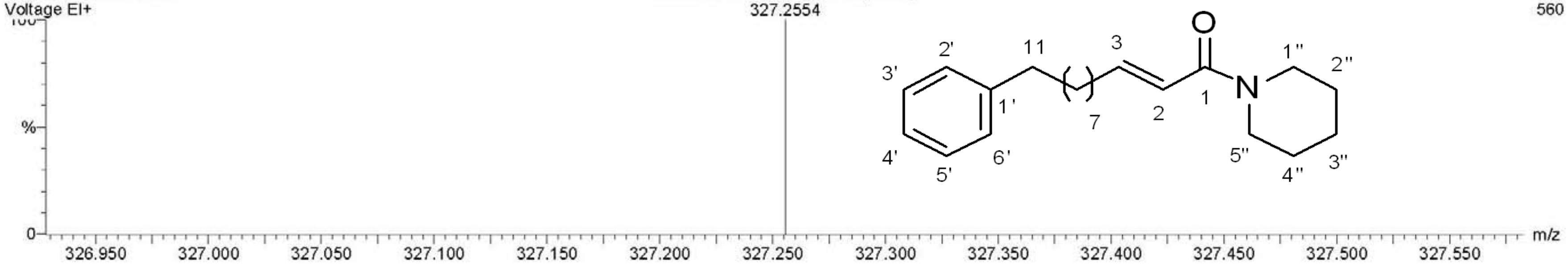

	Mass	Calc. Mass	mDa	PPM	DBE	i-FIT	Formula
Minimum:					-10.0		
Maximum:			100.0	10.0	120.0		
	327.2554	327.2562	-0.8	-2.4	7.0	5546293.5	C22 H33 N O

Display Report

Analysis Info

Analysis Name	D:\DATA\2012file\1210\121024\pcb23-41.d	Acquisition Date	10/24/2012 4:07:23 PM
Method	ms_ptservice.m	Operator	Bruker
Sample Name	pcb23-4	Instrument	HCT
Comment			

Acquisition Parameter

Ion Source Type	ESI	Ion Polarity	Positive	Alternating Ion Polarity	off
Mass Range Mode	Ultra Scan	Scan Begin	100 m/z	Scan End	900 m/z
Capillary Exit	16.0 Volt	Skimmer	40.0 Volt	Trap Drive	34.4
Accumulation Time	100000 µs	Averages	5 Spectra	Auto MS/MS	off

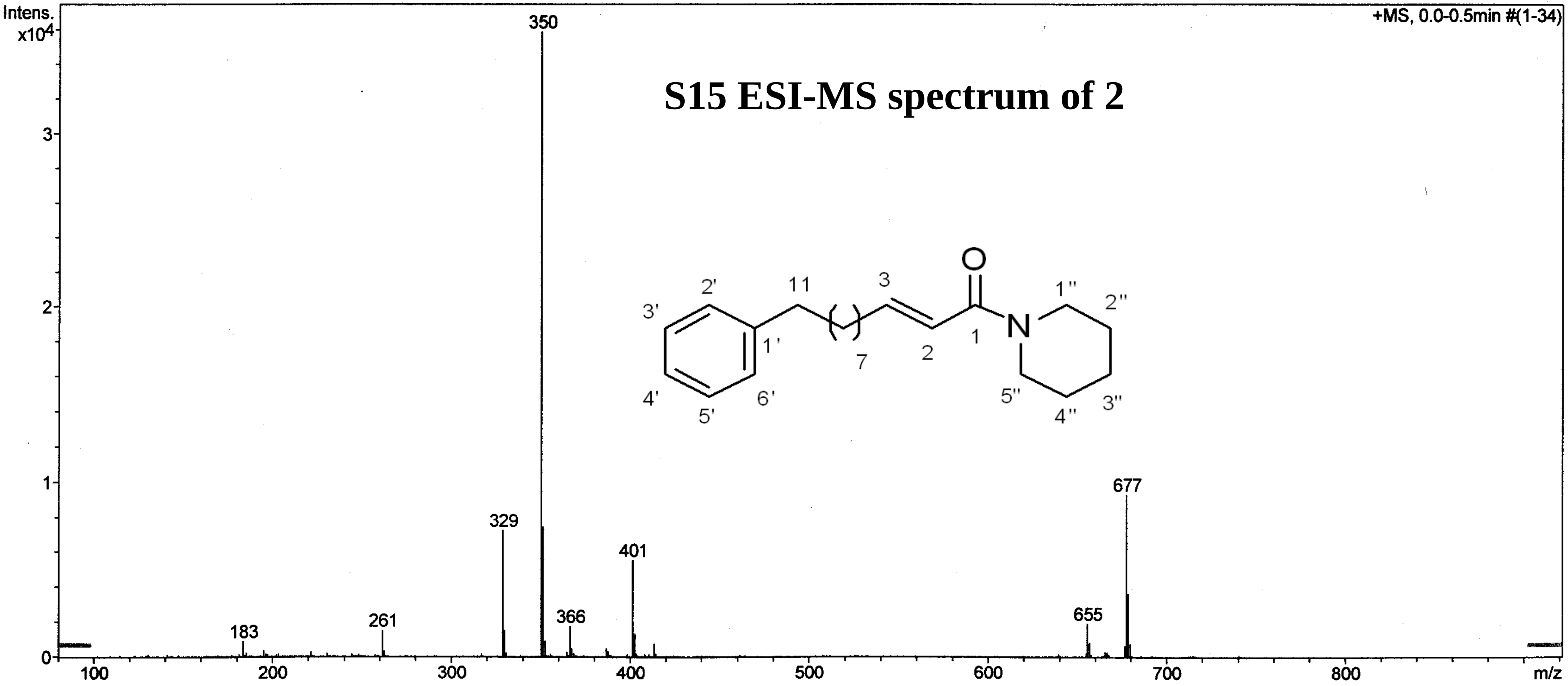

S15 ESI-MS spectrum of 2

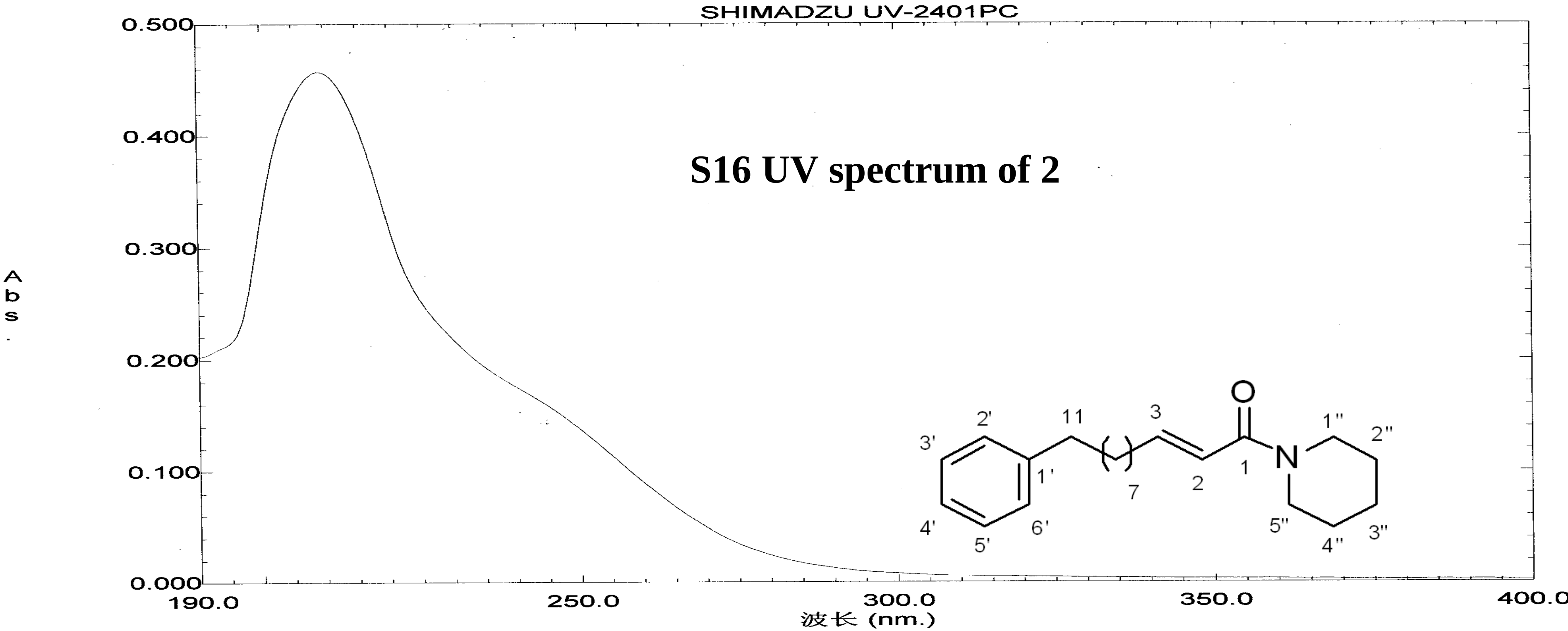

文件名: 13100906
样品名称: PCB234

创建于: 16:13 13-10-09
数据: 原始

测量模式: Abs.
扫描速度: 中速
狭缝: 5.0
采样间隔: 0.2

否.	波长 (nm.)	Abs.
1	209.20	0.4571

13100906

样品浓度: 0.0344毫克/毫升
溶剂: 甲醇

S17 ^{1}H NMR ($CDCl_3$, 600 MHz) spectrum of 3

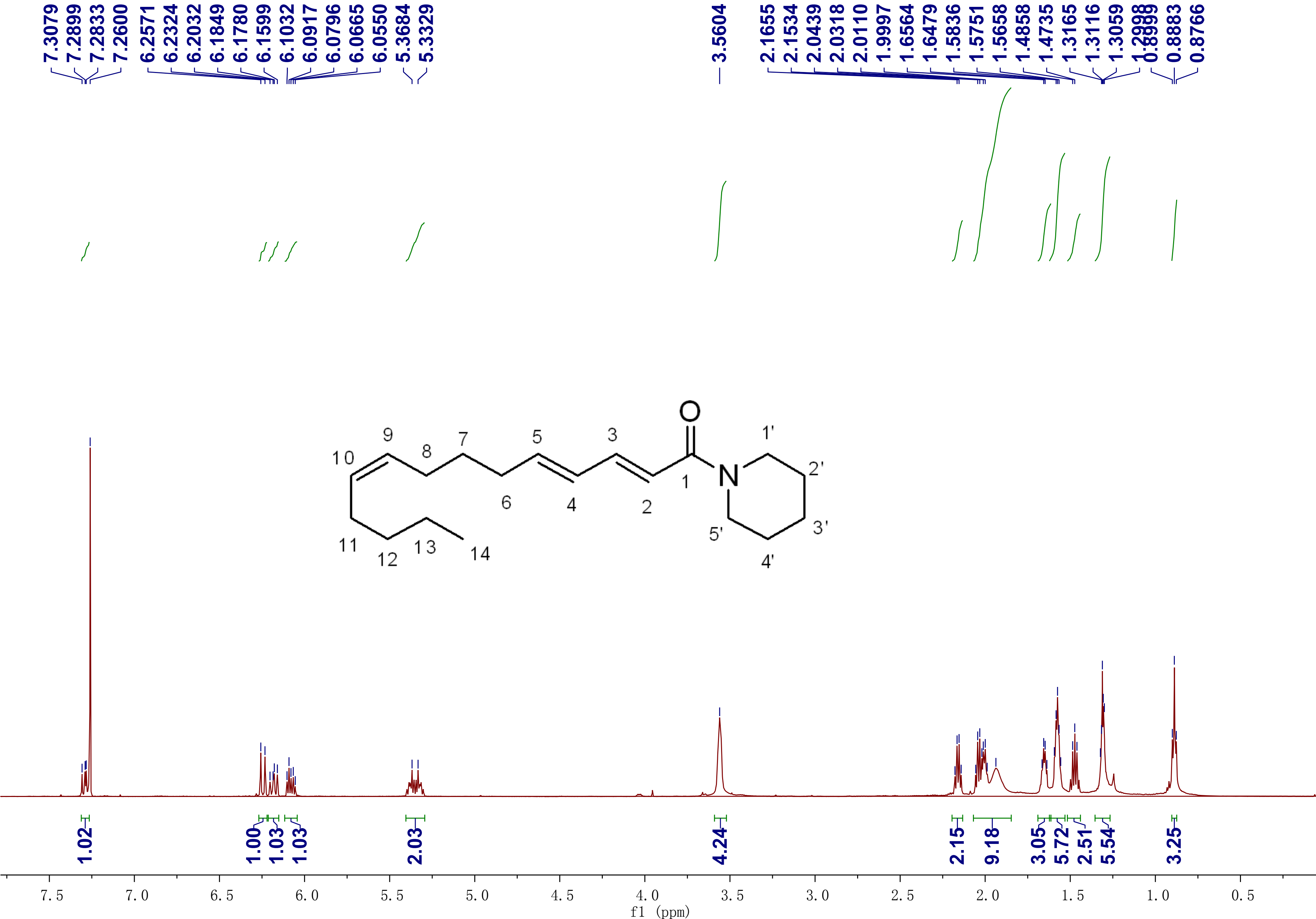

S18 ^{13}C NMR ($CDCl_3$, 150 MHz) spectrum of 3

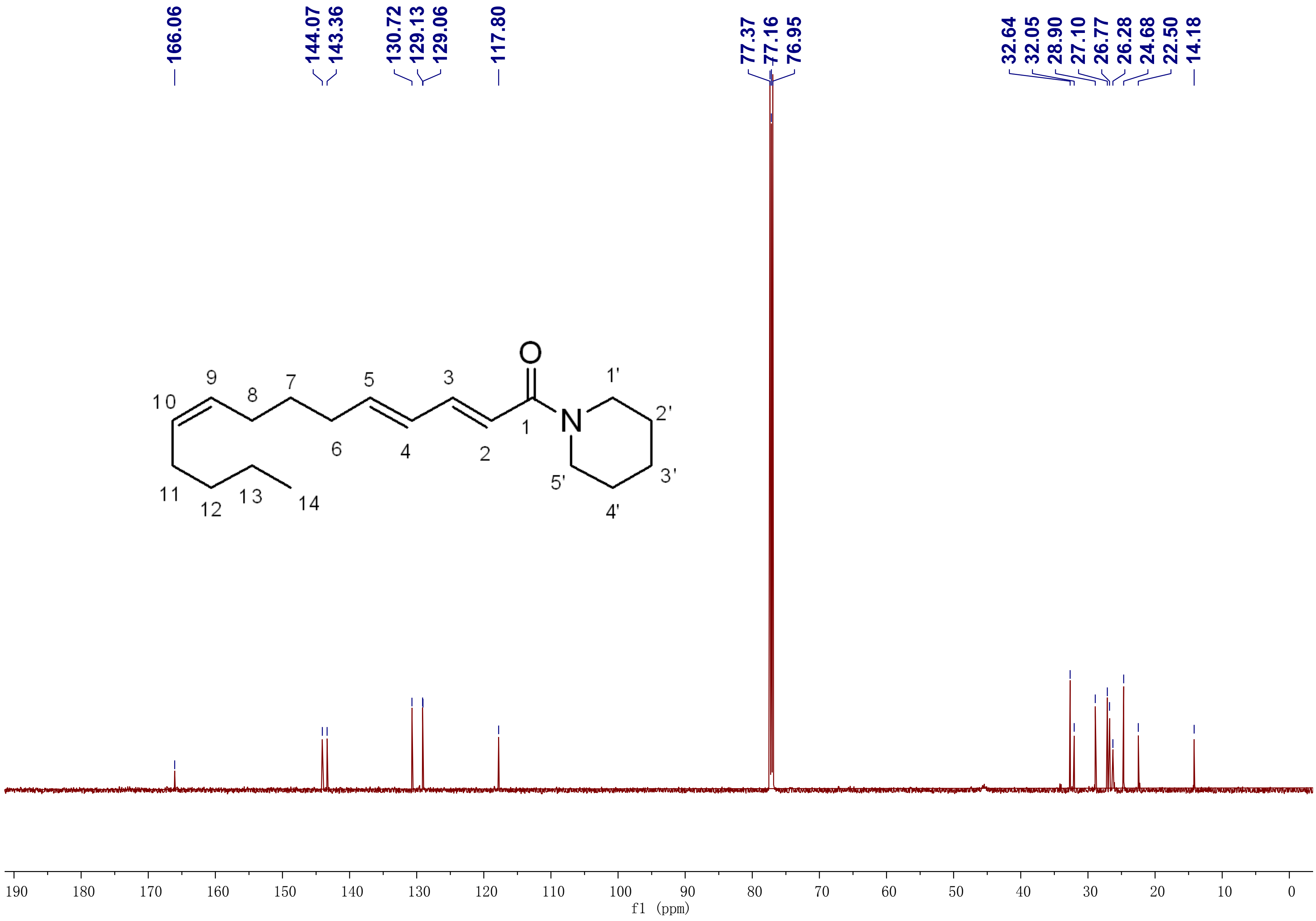

S19 HSQC spectrum of 3

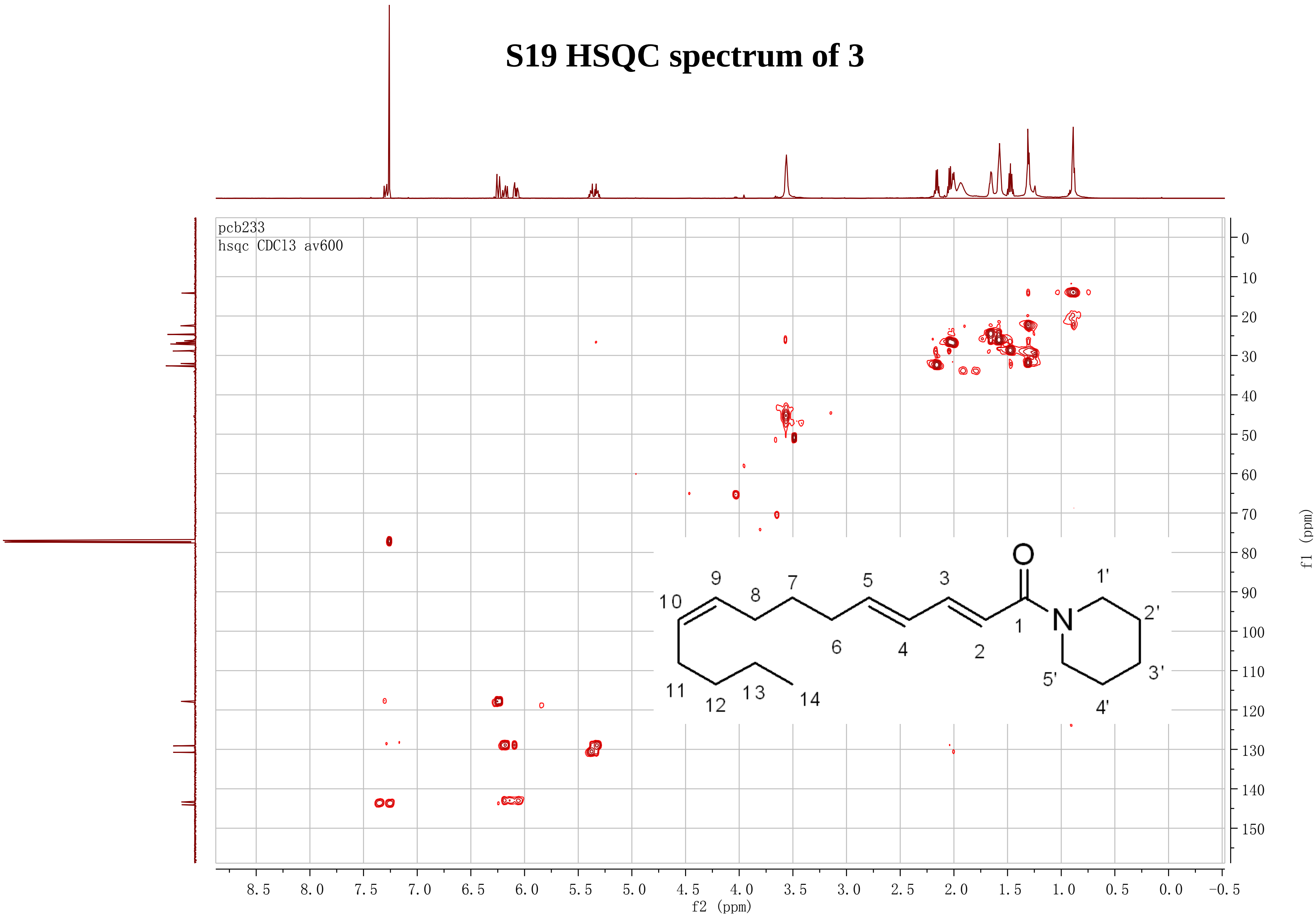

S20 HMBC spectrum of 3

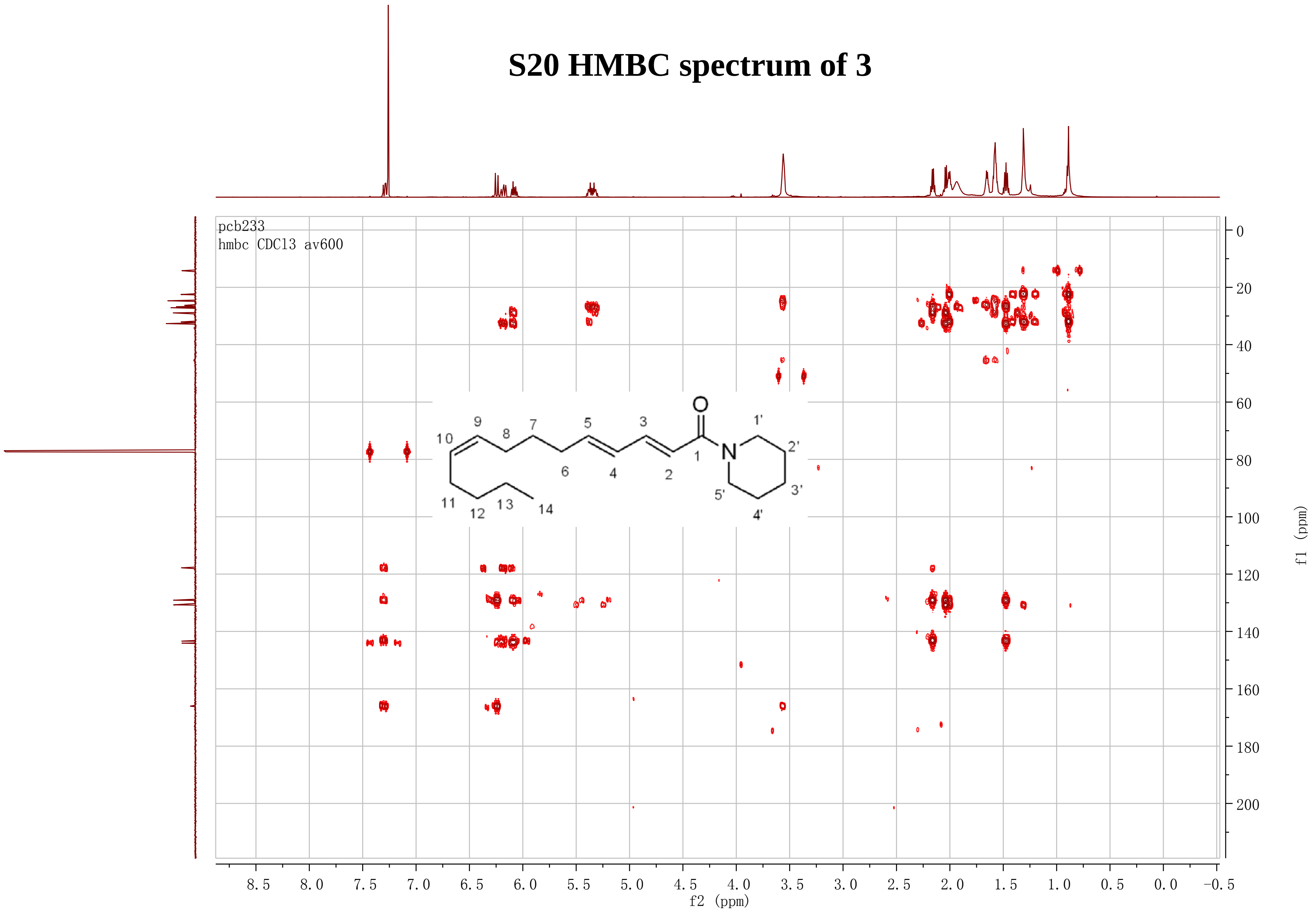

S21 ^{1}H-^{1}H COSY spectrum of 3

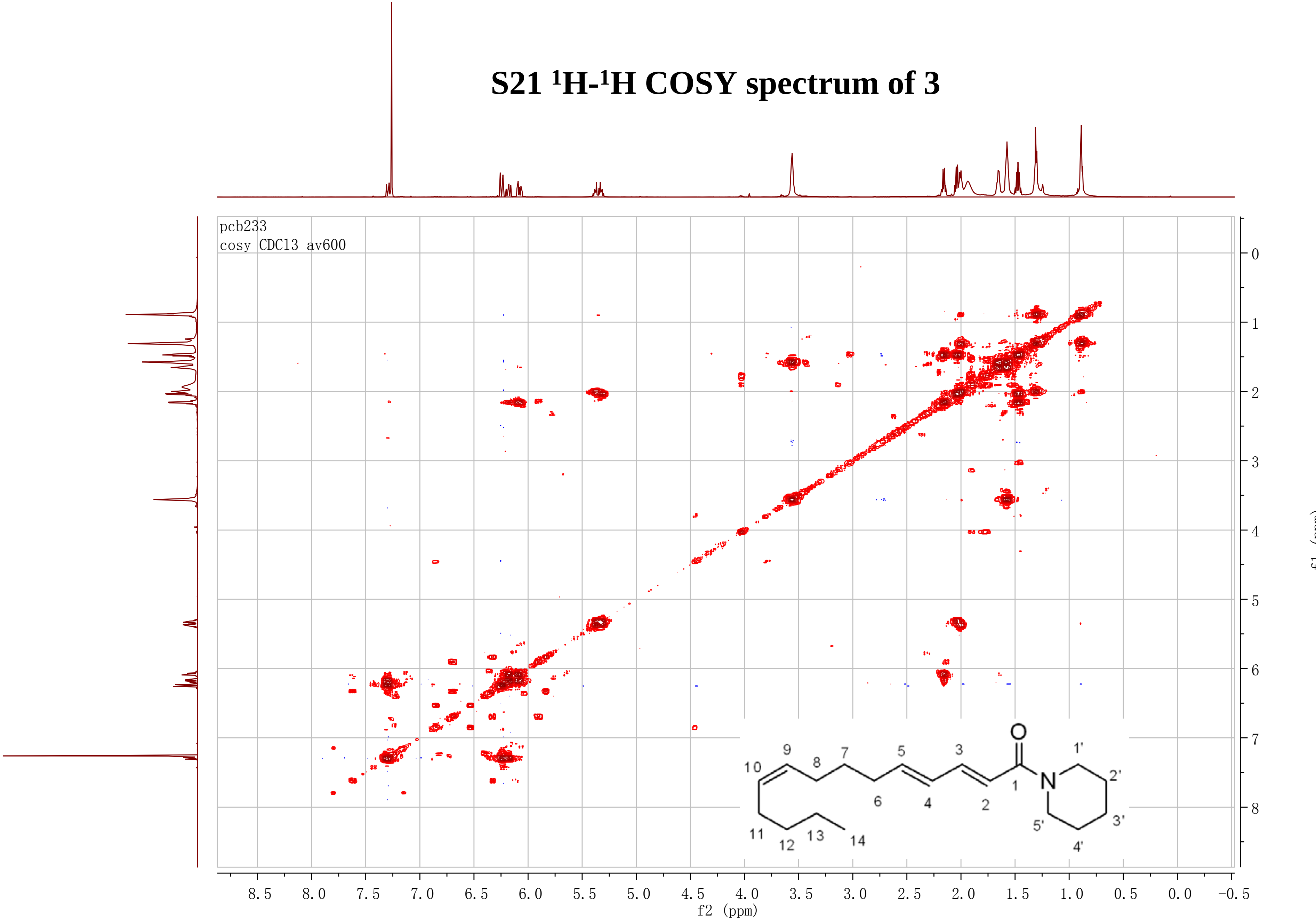

Elemental Composition Report

Single Mass Analysis

Tolerance = 10.0 PPM / DBE: min = -10.0, max = 120.0
Selected filters: None

S22 HREIMS spectrum of 3

Monoisotopic Mass, Odd and Even Electron Ions
16 formula(e) evaluated with 1 results within limits (up to 51 closest results for each mass)
Elements Used:
C: 0-200 H: 0-400 N: 1-1 O: 0-2

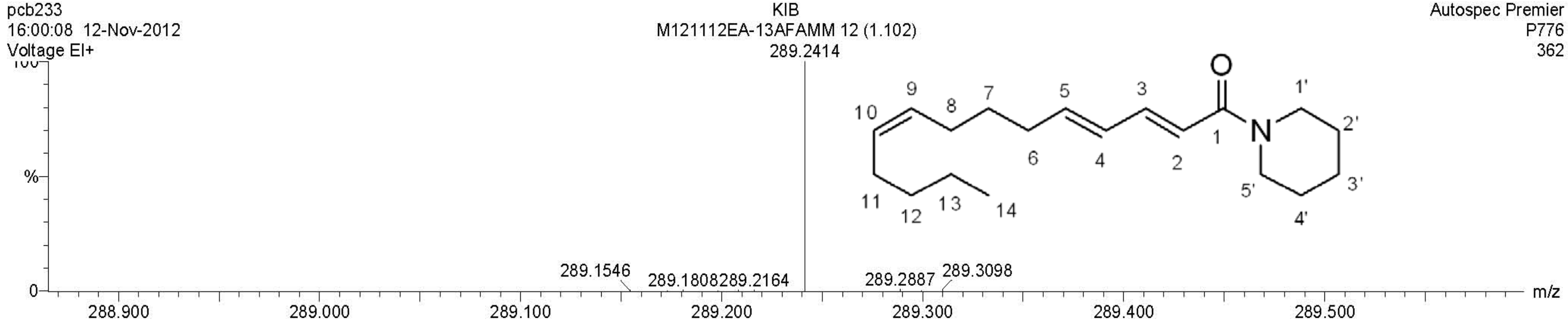

Minimum:				-10.0		
Maximum:		100.0	10.0	120.0		
Mass	Calc. Mass	mDa	PPM	DBE	i-FIT	Formula
289.2414	289.2406	0.8	2.8	5.0	5546192.0	C19 H31 N O

Display Report

Analysis Info

Analysis Name	D:\DATA\2012file\1210\121024\pcb23-30.d	Acquisition Date	10/24/2012 4:30:33 PM
Method	ms_ptservice.m	Operator	Bruker
Sample Name	pcb23-3	Instrument	HCT
Comment			

Acquisition Parameter

Ion Source Type	ESI	Ion Polarity	Positive	Alternating Ion Polarity	off
Mass Range Mode	Ultra Scan	Scan Begin	100 m/z	Scan End	1000 m/z
Capillary Exit	10.0 Volt	Skimmer	40.0 Volt	Trap Drive	30.4
Accumulation Time	100000 µs	Averages	5 Spectra	Auto MS/MS	off

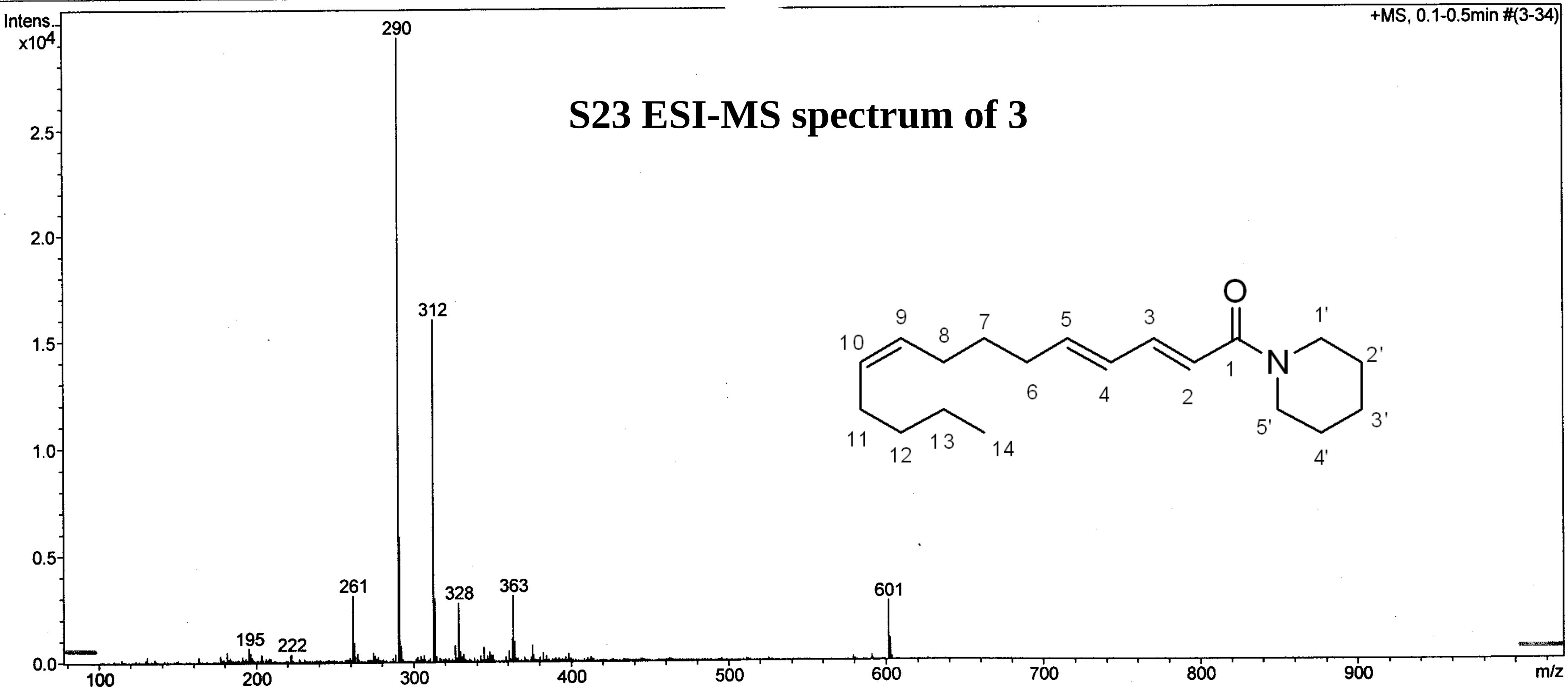

S23 ESI-MS spectrum of 3

S24 UV spectrum of 3

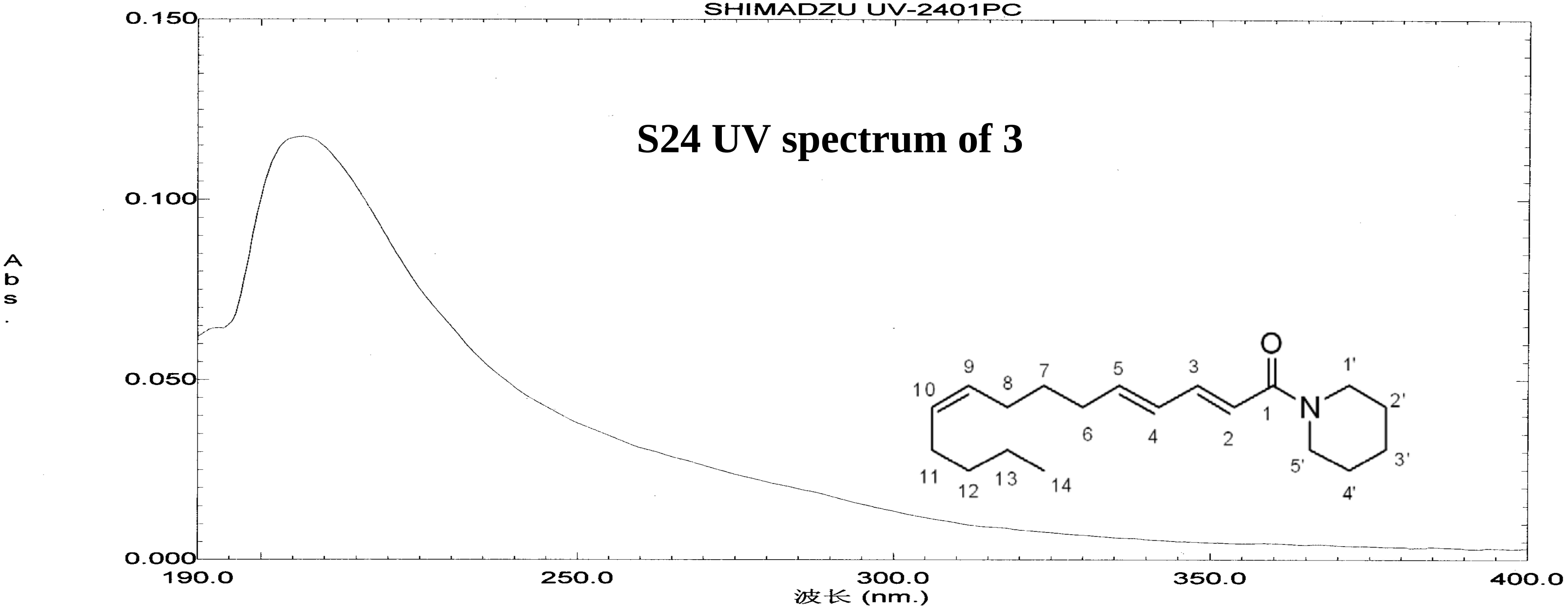

文件名:　13100905
样品名称：PCB233

创建于:　16:06　13-10-09
数据:　原始

测量模式:　Abs.
扫描速度:　中速
狭缝:　5.0
采样间隔:　0.2

否.	波长 (nm.)	Abs.
1	206.80	0.1174

13100905

样品浓度：0.0320毫克/毫升
溶剂：甲醇
